# The Recent Developments of Thermomechanical Processing for Biomedical Mg Alloys and Their Clinical Applications

**DOI:** 10.3390/ma18081718

**Published:** 2025-04-09

**Authors:** Hui Zhao, Jing Cheng, Chaochao Zhao, Min Wen, Rui Wang, Di Wu, Zhaoying Wu, Fang Yang, Liyuan Sheng

**Affiliations:** 1 School of Material Science and Engineering, Xi’an Shiyou University, Xi’an 710065, China; huier7921@126.com (H.Z.); 18220800985@163.com (J.C.); 2 PKU-HKUST ShenZhen-HongKong Institution, Shenzhen 518057, China; wenmin1027@foxmail.com (M.W.); wangrui833@163.com (R.W.); daisy_fangyang@163.com (F.Y.); 3 Shenzhen Institute, Peking University, Shenzhen 518057, China; cczhao_pkusz@yeah.net (C.Z.); wudi@ier.org.cn (D.W.); 4 School of Biomedical Engineering, Shenzhen Campus, Sun Yat-Sen University, Shenzhen 518107, China; 5 Shenzhen Airlines, Shenzhen Bao’an International Airport, Shenzhen 518128, China

**Keywords:** biomedical Mg alloy, thermomechanical processing, microstructure, mechanical properties, corrosion rate, cytocompatibility, clinical application

## Abstract

Magnesium (Mg) alloys have gained much attention for biomedical applications, due to their attractive properties, such as high specific strength, low density, low elasticity modulus, high damping capacity, biodegradation, and relatively good cytocompatibility. However, the biomedical use of Mg alloys also faces several challenges, primarily due to their low corrosion resistance and insufficient strength. Therefore, improving the strength and corrosion resistance of biomedical Mg alloys has become a critical issue. This review briefly summarizes the selection of appropriate alloying elements for biomedical Mg alloys, which is the fundamental factor in determining their microstructure, cytocompatibility, mechanical properties, and corrosion performance. It also discusses typical thermomechanical processing methods, including hot extrusion, hot rolling and hot forging, and examines the influence of deformation mode on microstructure, mechanical properties, and degradation behavior. Specifically, combining different thermomechanical processing methods could be an optimal choice, as it leverages the high efficiency and effectiveness of each method. Finally, the clinical application of biomedical Mg alloys in various fields are summarized and discussed to highlight their potential prospect and corresponding challenges. This review aims to provide insights for the rationale design and development of high-performance biomedical Mg alloys for widespread clinical applications.

## 1. Introduction

Magnesium (Mg), as one of the essential trace elements in the human body, second only to potassium, is often found in the form of Mg^2+^ in bone tissue and can be involved in many metabolic activities in the body, such as the activator of plenty of enzymes, muscle contraction, the transmission of neural excitability, the inhibition of abnormal excitation conduction, and so on [1,2,3,4,5]. Moreover, Mg is also thought as a promising biomedical material due to its attractive cytocompatibility and biodegradability [6,7,8]. Unlike other metal materials, Mg degrades in the body in a biologically safe manner, releasing non-toxic ions and byproducts of Mg(OH)_2_ and hydrogen gas, which have minimal impact on the human body [6,9,10,11]. As a result, Mg demonstrates great potential for application in many medical fields such as orthopedics, cardiovascular, oral, gastrointestinal, oncology, and others [12,13,14]. Despite these advantages, the Mg still has to face many challenges such as limited mechanical strength and insufficient corrosion resistance in the body environment [10,15]. To conquer these problems, many methods have been applied, among which alloying and thermomechanical processing have been thought of as the most convenient and effective way [16,17,18]. Therefore, many kinds of Mg alloys such as Mg-Zn-based and Mg-RE (rare earth)-based alloys have been developed, while the corresponding thermomechanical processing of hot extrusion, hot rolling, and hot forging have been developed as well.

Generally, the alloying composition and thermomechanical processing fundamentally determine the mechanical properties and corrosion resistance of Mg alloys, primarily through grain refinement, solid solution, precipitation, and crystal defects [6,19,20]. Specifically, the design of the alloy composition dictates the type and proportion of second phases, while subsequent thermomechanical processing further optimizes the grain structure of the matrix, as well as the size and spatial distribution of secondary phases. Consequently, the strategic design of alloy compositions, coupled with appropriate processing techniques, provides a promising way to improve the performance of biomedical Mg alloys [1,21,22,23]. Furthermore, for a specific Mg alloy, optimizing the processing method and parameters can significantly improve the microstructure and performance, thereby enhancing its service life.

This review provides a comprehensive and up-to-date overview of biomedical Mg alloys, with a focus on alloying composition selection, thermomechanical processing methods, and clinical applications. This review emphasizes design strategies and recent advancements, including typical examples. We begin by outlining the development of biomedical Mg alloys, summarizing its advantages or challenges, alloy composition selection, and performance requirements in Section 1. Section 2 focuses on the development of thermomechanical processing for the improvement of mechanical properties and corrosion resistance. The clinical application and exploration of biomedical Mg alloys are summarized in Section 3. Finally, Section 4 provides conclusions and perspectives, addressing current limitations, challenges, and potential solutions. This review aims to provide valuable insights for the development of high-performance biomedical Mg alloys for new biodegradable implants.

### 1.1. Background of Biomedical Mg Alloys

Because of attractive advantages such as high specific strength, high damping capacity, high specific strength, exceptional impact resistance, and bioactive performance, the Mg alloy has garnered widespread interest in many fields [24,25]. Moreover, its low density (1.7–2.0 g/cm^3^), elastic modulus closely resembling that of human bone (41–45 GPa), biodegradability, and cytocompatibility features make the Mg alloy a highly expected candidate for biomedical implants [9,26,27]. Due to its activity in biological environments, the Mg alloy would experience gradual degradation, and its byproducts would be eliminated by absorbing or by metabolic processes, thereby avoiding secondary surgical removal like conventional metal implants [28,29]. Actually, the released ions during degradation could exhibit some function, such as osteogenesis promotion and anti-tumor, which endows Mg alloys more expectation [24,30,31,32]. Despite the attractive advantages of Mg alloys in biomedical applications, their non-ideal corrosion resistance and mechanical properties within biological environments handicap their widespread application [33,34]. As a kind of material for biomedical applications, cytocompatibility and biosafety are the basic requirements, which demand sufficient mechanical support and the non-toxicity of degraded products. Currently, the in vivo corrosion rate of Mg alloys remains a great challenge to regulate, and the potentially excessive degradation would compromise their capability for long-term structural support [35,36]. As a result, the uncontrollable corrosion behavior of Mg alloys also affects the reliability of their implants, resulting in unexpected failure [37,38]. Thus, it is a critical issue for biomedical Mg alloys to balance the mechanical properties and corrosion performance.

To solve these challenges, innovative strategies were developed in succession to enhance the corrosion resistance of biomedical Mg alloys. The main attempts performed now are the optimizing of alloy composition and the corresponding processing methods or parameters. In general, the microstructure, phase morphology, and crystal defects can be well regulated, which contributes to the mechanical properties, corrosion behavior, and cytocompatibility simultaneously. It is anticipated that the improved performance of Mg alloys could extend their application.

### 1.2. Design of Chemical Compositions for Biomedical Mg Alloys

For biomedical Mg alloys, the primary requirement is safety and effectiveness during their implantation in the human body. Correspondingly, the toxicity of alloying elements, degradation rate, degradation behavior, the metabolism of degraded products, mechanical properties, and so on should be qualified for tissue repair [39,40]. Therefore, the research and development of biomedical Mg alloys must holistically address these demanded performance. First and foremost, mechanical properties are paramount, including strength and toughness under physiological loading conditions, alongside the elastic adaptation with repairing hard tissues. Secondly, the cytocompatibility demands that the Mg alloys to be adapted to cellular processes, including growth, adhesion, and metabolism. Then, the effectively controlled corrosion behavior is critical, as rapid and inhomogeneous degradation both result in premature failure, compromising tissue regeneration or healing. During the design of biomedical Mg alloys, the selection of chemical compositions should fully consider their effect on mechanical, physical, and biological properties [41,42,43].

Recently, the Mg alloys have been applied to develop several kinds of clinical implants such as intramedullary nails, hemostatic clips, cardiovascular stents, skin sutures, bone plates, and screws [44,45,46,47,48,49,50]. Due to the different application environments, these implants impose different requirements on adopted Mg alloys [48]. An intramedullary nail should have sufficient mechanical strength to meet the requirement in bending resistance, providing reliable internal fixation [45]. For the bone fixation surgery, the bone plate and screw have to bear the long-term load of the hard tissue, such as tensile, compressive, shear, and torsional stresses, which requires higher strength for selected alloys [51,52,53,54]. Conversely, hemostatic clips demand high plasticity to obtain the clamping effect and occlusion capacity, maintaining short-term structural integrity to effectively prevent postoperative bleeding [47,49,50]. A skin staple mainly requires the Mg alloy to have high toughness cytocompatibility and plasticity to form secure knots [47]. Comparatively, a cardiovascular stent requires a balanced performance between radial support and vascular compliance, which is crucial to focal vascular repairing [52]. Only the Mg alloy that has high strength and ductility could meet such requirements. Specifically, the functional demands of these implants determine the selection of the Mg alloy. Hence, the design of a biomedical Mg alloy with an appropriate chemical composition is critical to meet the specific functional requirements of various medical implants.

Combined with the principle of alloy smelting, the bioactive and non-toxic metallic elements would be the priority selection for additives in the designed Mg alloys. The alloying elements in Mg alloys precisely exert a critical influence on its corrosion behavior and mechanical performance [55,56]. The following factors should be considered for the designed biomedical Mg alloy, including the specific application field (bone fixture, vascular repair, soft tissue repair, etc.), functional requirements (osteointegration, anti-bacteria, etc.), phase formation (kinds, morphology, distribution), performance (corrosion, strength, plasticity, etc.), and processing strategies (rolling, extrusion, forging, etc.). These factors help to construct the alloy design principles, which contribute to realizing the balance among chemical, physical, mechanical, and biological properties [57,58,59]. Except for the alloying elements, there is some impurities required to be strictly controlled in Mg alloys, since they extremely decrease mechanical properties, corrosion performance, and cytocompatibility [60,61]. Currently, extensive studies have been conducted on biomedical binary Mg alloys, including Mg-Zn, Mg-Ca, Mg-RE, Mg-Mn, Mg-Sn, Mg-Sr, Mg-Li, and Mg-Cu. In particular, the inclusion of rare earth (RE) elements can enhance the performance of biomedical Mg alloys through various mechanisms. As a result, several RE elements have been incorporated, such as yttrium (Y), neodymium (Nd), cerium (Ce), lanthanum (La), gadolinium (Gd), and dysprosium (Dy) [19,62,63,64]. Based on the binary alloys, more multi-component alloys are developed with specific applications. However, the effects of the alloying elements should be fully considered during the alloy design process, by which the performance could be presupposed and optimized beforehand. A summary of the biological and performance benefits of alloying elements for biomedical Mg alloys is given in Table 1.

### 1.3. Performance of Biomedical Mg Alloys by Alloying Composition

Due to the fundamental effect of alloying, extensive improvements have been achieved in Mg alloys, especially in mechanical strength and corrosion resistance [65,66,67,68,69,70,71,72,73]. Generally, the addition of alloying elements in Mg alloy always induces the formation of a secondary phase, which basically contributes to strength and hardness. The simultaneous grain refinement can benefit ductility, if the secondary phase has a dispersed distribution and small size. Due to the high corrosion potential of most alloying elements, they can primarily enhance the corrosion resistance of Mg alloy, but the formed galvanic reactions are detrimental for corrosion resistance, which should be balanced during alloy design. Even though the suggested alloying elements have good compatibility, they always have content limitations [70,74]. Some alloying elements would exhibit toxicity to the human body, when their concentrations exceed specific values. As a result, the type and content of alloying elements should be considered simultaneously during the alloy design [70,71,72,73]. Although alloying is an effective approach to enhance the mechanical properties and corrosion resistance of Mg alloys, its application in biomedical Mg alloys remains constrained, as it fails to fully satisfy the dual requirements of cytocompatibility and performance simultaneously.

To overcome this challenge, thermomechanical processing technologies have been well adopted as a pivotal strategy to improve the performance of biomedical Mg alloys [75,76,77]. These techniques cooperated with alloying, significantly enhancing the mechanical properties, corrosion resistance, and cytocompatibility of Mg alloys by refining their grain size, tailoring the microstructure, and regulating inner crystal defects [78,79]. Among these, extrusion, rolling and forging techniques play an important role in improving the strength, hardness, ductility, and corrosion resistance of Mg alloys [80,81,82]. For instance, Kiani et al. [80] demonstrated that the extrusion process effectively optimized the distribution of alloying elements and facilitated favorable texture formation in Mg-Zr-Sr-Dy alloys, thereby significantly enhancing tensile and compressive properties while concurrently improving corrosion resistance and cytocompatibility. Deng et al. [83] fabricated RE-doped Mg alloy sheets using multiple passes of small-reduction rolling, which revealed the rolling process and not only substantially enhanced the strength and ductility of the RE-doped Mg alloy but also addressed its morphological application constraints. Zou et al. [82] demonstrated, through an integration of numerical simulations and experimental validation, that radial forging effectively refines grain structures and enhances tensile strength and ductility. Consequently, the overall mechanical performance of the ZK60 alloy has been improved significantly. In general, the synergistic integration of alloying and processing technologies offers technical support for the optimization of biomedical Mg alloys.

In summary, deformation processing has markedly enhanced the overall properties of biomedical Mg alloys, particularly for mechanical properties [81]. Actually, the microstructure refinement by deformation also weakens the galvanic reaction and helps to improve corrosion resistance and cytocompatibility as well. Furthermore, employing appropriate processing techniques could eliminate the initial metallurgic defects and regulate the microstructure, which benefits the homogeneous degradation and its reliability as biomedical implants [80]. Therefore, this paper aims to systematically review the primary processing techniques for biomedical Mg alloys in recent years and their contribution to corresponding biomedical applications. By conducting an in-depth analysis of existing research, it is anticipated to offer robust theoretical and practical insights, facilitating the development of biomedical Mg alloys.

## 2. Deformation for Biomedical Mg Alloy

For the Mg alloy, its high chemical activity always leads to the formation of impurities during its metallurgical processing, which is detrimental to its performance and service life [79,82]. To address these challenges, thermomechanical processing has been well applied to Mg alloys. The rheological behavior of Mg alloys caused by the deformation of thermomechanical processing optimizes the microstructure obviously by homogenizing structures, reducing porosity, refining grains, fragmenting large second-phase particles, and inducing high-density dislocations [82,84]. Notably, fine-grained strengthening, a primary strengthening mechanism in metallic materials, would play an important role in improving the mechanical properties of Mg alloys [84]. Additionally, grain refinement also contributes to corrosion resistance by eliminating bulk secondary phases, achieving homogeneous degradation and a low corrosion rate [85]. Furthermore, a fine-grain microstructure facilitates the rapid formation of a relatively dense and stable passivation layer on the alloy surface, thereby further enhancing corrosion resistance. However, to realize these improvements, the precise control of processing parameters is necessary, because these factors influence the formation of texture and dislocation, alongside secondary phase morphology [86]. Within the fine-grain-size regime, the decreased grain size weakens the typical preferential corrosion effect of grain boundaries during the initial stage, since the rapidly formed passivation layer is induced by the fine grain structure. The formed crystal defects promote homogeneous corrosion, which restrains extreme local corrosion and benefits corrosion resistance [87].

With optimized deformation processing, the microstructure, mechanical properties, and corrosion performance of biomedical Mg alloys could be well improved. Specifically, thermomechanical processing, such as extrusion, rolling, and forging, always has its individual deformation features, which endows the specific properties on the processed Mg alloy. The detailed advantages and disadvantages of different thermomechanical processing techniques are listed in Table 2. This section mainly focuses on the widely adopted processing techniques for the deformation of biomedical Mg alloys, demonstrating their features and evaluating potential challenges.

### 2.1. Processing Technologies for Biomedical Mg Alloys

It is well known that Mg alloys possess a hexagonal close-packed (hcp) crystal structure. The relatively low symmetry of this crystal structure, compared to the cubic system, constrains the processing performance of Mg alloys at room temperature, necessitating thermomechanical processing to obtain great plastic deformation [85,107,108,109]. Thermomechanical processing could take full use of the improved deformability of Mg alloys at high temperatures to realize the rheological deformation, rendering them better suited to stringent forming demands [108]. Hot extrusion, hot rolling, and hot forging are widely employed traditional techniques for the processing of biomedical Mg alloys. By different rheological deformations, the grain size, secondary phase morphology, and crystal defects of the biomedical Mg alloy would be fully regulated, by which its mechanical properties, cytocompatibility, and corrosion resistance are optimized as well [78]. Such improvements help to meet the stringent requirements of biomedical implants, especially for orthopedic implants [84].

Due to its specific constrained forming feature, hot extrusion is the primary processing method for biomedical Mg alloys. The common hot extrusion technique has different modes, including conventional extrusion (direct extrusion), equal-channel angular pressing, and cyclic extrusion compression [91,110]. Conventional extrusion (direct extrusion) is the most extensively employed method for the deformation of biomedical Mg alloys, which significantly enhances the mechanical properties of biomedical Mg alloys by mitigating casting defects, refining grain structures, dispersing secondary phases, weakening basal textures, and activating non-basal slip systems [76]. In general, the extrusion temperature, extrusion velocity, and extrusion ratio play critical roles, which determines the microstructure, texture, and mechanical properties of biomedical Mg alloys [111,112].

Different from the constrained deformation of hot extrusion, hot rolling has more freedom, which mainly controls the sectional area between rollers [113]. Such a feature helps to achieve a high deformation rate by one-pass or multi-pass rolling [114]. Thus, hot rolling is widely utilized to process biomedical Mg alloys to obtain highly accumulated deformation and includes conventional rolling, cross-rolling, and accumulative roll bonding. Notably, conventional rolling is extensively employed because of its convenience and effectiveness. For instance, Guan et al. [112] successfully developed a biodegradable Mg-Zn-Sr alloy via conventional rolling, demonstrating remarkable enhancements in both mechanical properties and corrosion resistance.

Compared to hot extrusion and hot rolling, hot forging is relatively underutilized in the domain of biomedical Mg alloys. Nevertheless, hot forging has distinct advantages, including the elimination of casting defects and an optimized microstructure with less texture, which contributes to strength and ductility simultaneously [115,116]. Moreover, the high freedom deformation of hot forging is more suitable for the initial processing of biomedical Mg alloys [82]. To enhance the comprehensive properties of biomedical Mg alloys, researchers could employ multi-directional forging and radial forging (rotary swaging) techniques to obtain better microstructure and mechanical performance. Martynenko et al. [117] investigated ZX11 Mg alloys subjected to rotary swaging (radial forging) and demonstrated that this technique significantly refines grain structure, enhancing strength, ductility, and fatigue strength simultaneously. Furthermore, rotary swaging processing has almost no adverse impact on the corrosion resistance of Mg alloys, which provides a practical method for improving biomedical Mg alloys. However, the processing efficiency of radial forging and rotary swaging is really low, which limits its wide application.

#### 2.1.1. Hot Extrusion

Hot extrusion refers to the extrusion of a metallic material at a certain temperature, where the billet is heated above the recrystallization temperature prior to extrusion [118]. Hot extrusion has numerous advantages, including simple operation, high dimensional accuracy, small machining allowances, and high surface finish. Furthermore, hot extrusion can also be applied to process low-plasticity Mg alloys, using back pressure or capsuling [118,119]. Therefore, hot extrusion is almost the preferential choice for the processing of biomedical Mg alloys.

##### Conventional Extrusion (Direct Extrusion)

**Figure 1:**
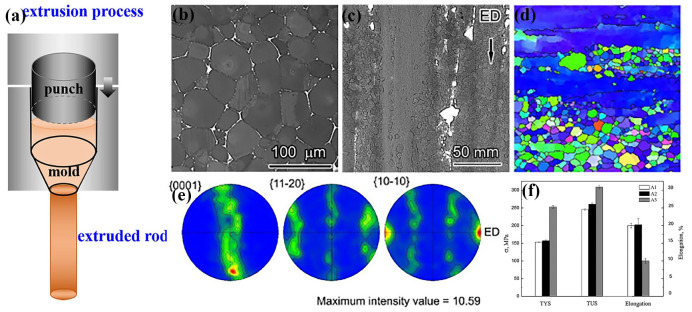
(**a**) Schematic diagram of the extrusion process [120]; (**b**,**c**) SEM image of the (**b**) as-cast and (**c**) extruded Mg-Zn-Y-Nd alloys (Arrow indicating extrusion direction, ED); (**d**,**e**) EBSD results of extruded Mg-Zn-Y-Nd alloys: (**d**) inverse polar figure, (**e**) polar figure; and (**f**) tensile and compressive properties of the hot extruded Mg-Zn-Y-Nd alloys [123].

The schematic diagram of the conventional hot extrusion (direct extrusion) processing is shown in Figure 1a [120]. This kind of extrusion realizes the deformation of biomedical Mg alloys by extruding it in a mold with straight and taper cavities. The extrusion ratio could be regulated by changing the final size of the tapered cavity, and the extrusion speed could be changed by adjusting the force exerted on the punch and its moving speed. During the extrusion, the force direction is on the same axis as the extrusion direction, which indicates the turbulent deformation in the taper cavity region. Nevertheless, the simple rheological deformation results in the linear feature of the grain morphology along the extrusion direction and typical textures inside. Previous studies have established that the parameters of conventional extrusion could significantly influence the grain size, secondary phase morphology, texture, substructure, and dislocations, thereby regulating the mechanical properties and corrosion resistance of biomedical Mg alloys [80,107,121,122]. Du et al. [123] conducted a comprehensive investigation on the Mg-Zn-Y-Nd alloys prepared by conventional extrusion and demonstrated the dual-size fine grain structure, prominent fiber texture, linearly distributed secondary phases, and diversified distribution of substructures (Figure 1b–e). Such microstructure indicates the different mechanical properties in axial and radial directions. The increased extrusion ratio enhanced strength but decreased ductility (Figure 1f). Clearly, accompanying grain refinement, the greatly increased dislocation density, substructure, and texturing would be detrimental to ductility.

During the conventional extrusion of biomedical Mg alloys, temperature is an important factor that determines the plasticity. The typical extrusion temperature for the biomedical Mg alloy that was scoped is from 300 °C to 470 °C, while the extrusion ratio generally ranges from 5:1 to 100:1 [6]. For the specific biomedical Mg alloy, its extrusion temperature could be under 300 °C, which benefits the grain refinement [66,107]. In general, the main parameters for conventional extrusion include extrusion temperature, extrusion ratio, and extrusion speed. A higher extrusion temperature results in a coarser microstructure, while a higher extrusion ratio results in a finer microstructure. The extrusion speed mainly affects the shape of the extruded billet and substructures inside. Then, the appropriate assembly of extrusion parameters becomes so important that it directly determines the microstructure and mechanical properties of the biomedical Mg alloy [111]. The research revealed that the extrusion at a relatively low temperature contributed to grain refinement and dislocation formation, obviously improving strength [124]. Conversely, extrusion with higher temperatures benefited recrystallization and grain growth, which increased ductility and corrosion resistance [107,124]. More investigations demonstrated that higher extrusion ratios facilitated dynamic recrystallization (DRX), but the great deformation led to plenty of substructures and dislocations, which was harmful to ductility. The increasing extrusion passes with a small extrusion ratio could realize the fine-grain structure and restrain the DRX, which would benefit the strength and ductility simultaneously [125]. Moreover, simultaneous increases in the extrusion ratio and extrusion passes resulted in the significant refinement of grains and secondary phases, further improving microstructure uniformity and deforming coordination [126]. The over-increase in the extrusion ratio might lead to an obvious reduction in the ductility of biomedical Mg alloys, except for the markedly enhanced strength. However, the extrusion with more extrusion passes and a relatively small extrusion ratio requires the initial billet with a big diameter, which also increases the fabrication difficulty. In general, the simple extrusion mold structure results in the complex synergy of parameters, which increases the difficulty of achieving an ideal microstructure and mechanical properties [6,123,125,126,127].

To address the inherent limitations of conventional extrusion, advanced methods such as equal-channel angular pressing (ECAP) and cyclic extrusion compression (CEC) have been innovatively developed.

##### Equal-Channel Angular Pressing

**Figure 2:**
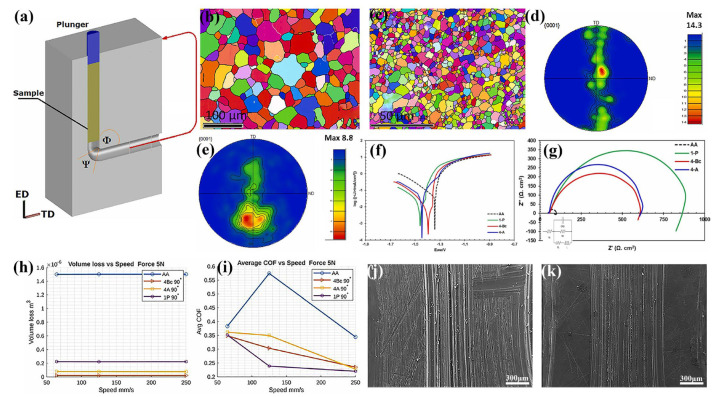
(**a**) Schematic of the ECAP process; (**b**,**c**) EBSD orientation maps for the ZK30 relative to ND for the (**b**) annealed ZK30 and (**c**) post–ECAP processing over 4A (with no rotation between the subsequent passes up to 4 passes) [129]; pole figures of the Mg alloy in its (**d**) annealed condition and (**e**) ECAP–processed through 4A; (**f**) PDP curves and (**g**) the Nyquist plot of ZK30 alloy; volume loss of the ZK30 alloy (**h**) and the average coefficient of friction at a force of 5 N (**i**); and the worn surface micrographs of the ZK30 alloy after the wear test: (**j**) annealing alloy, (**k**) ECAP-processed [128].

Different from the conventional extrusion, equal-channel angular pressing (ECAP) realizes the uniform grain refinement using the intense shear deformation in the connecting corner with an angle of about 90°, as shown in Figure 2a [128]. With such a deformation mode, ECAP possesses distinct advantages. Firstly, the biomedical Mg alloy processed ECAP could have a refined and homogenized microstructure, due to the shear deformation. Consequently, the strength and ductility could be enhanced simultaneously [129,130,131]. Due to the intense deformation, ECAP could regulate the texture of the initial alloy, which improves the mechanical properties. Especially, this could be used to increase the isotropic performance, benefiting the corrosion resistance under specific stress [132]. In addition, the great deformation during ECAP could promote the DRX and eliminate some crystal defects, contributing microstructural stability [133]. Because of the equal channel feature, the biomedical Mg alloy could be processed by the ECAP repeatedly to obtain an ultra-fine microstructure and higher strength [88,128].

Because of the deformation feature of the ECAP process, the biomedical Mg alloy experiences substantial shear deformation while retaining its original cross-sectional dimensions. Such a deformation would induce considerable strains inside and modify the microstructure significantly [134]. Alateyah et al. [135] observed that ECAP markedly enhanced the DRX in the alloy, which contributed to the formation of texture. During the ECAP process, critical parameters such as the ECAP passes and processing temperature exert great influence on microstructural evolution and mechanical performance [134,136]. When the ECAP process is conducted within the temperature range of 180 °C to 450 °C, the elevating temperature suppresses twin formation and facilitates dislocation slip, which increases dislocation density significantly [136]. Correspondingly, the microstructure and grain orientation of the alloy could be well regulated, achieving the initial set objective.

Due to the accumulation of stress in the processed alloy, there is a threshold value for ECAP, whose change in processing temperature or passes might lead to extensive internal cracking and a fragmented secondary phase in DRX grains [137]. Such a threshold value of the ECAP is contingent upon the specific Mg alloy compositions and associated processing parameters [135,138,139]. For instance, Arhin et al. [138] achieved full dynamic recrystallization through 16 ECAP passes, producing an ME21 Mg alloy characterized by a homogeneous and ultra-fine grain structure. Li et al. [139] conducted eight ECAP passes processing the Mg-Zn-Mn-Ca alloy, obtaining a microstructure with finer and more uniform grains, compared with the extruded state. Consequently, the alloy had highly improved corrosion resistance in simulated body fluid. Subsequent investigations demonstrated that the wear resistance of the Mg alloys could be enhanced by ECAP, due to the greatly increased hardness [128,140]. Alateyah et al. [128] investigated the Mg-3Zn-0.6Zr (ZK30) alloy with four passes of ECAP processing and revealed that its microstructure and texture had experienced pronounced evolution, which obviously affected wear and corrosion resistance. The EBSD analyses revealed that ECAP processing induced a remarkable 92.7% reduction in grain size (Figure 2b,c) alongside a greatly weakened texture (Figure 2d,e). By the ECAP processing, the ZK30 alloy exhibited significantly enhanced corrosion resistance (Figure 2f,g) and markedly improved wear performance (Figure 2h–k). A comparative analysis of the wear surfaces of the annealed ZK30 alloy and the processed ECAP revealed that the ECAP decreased the width of the wear groove obviously, further verifying the improving effect of ECAP in the wear resistance of Mg alloys.

ECAP offers a different microstructural regulation mode for the biomedical Mg alloy, demonstrating distinct advantages in grain refinement. The accompanied DRX benefits the gain size homogenizing and improves strength, ductility, corrosion, and wear resistance simultaneously [141,142,143,144]. However, the intense shear deformation indicates its size limitation for the processed alloy, which significantly restrains the application of this kind of technique for the processing of bulk Mg alloys. Therefore, ECAP could be used to fabricate the biomedical Mg alloy bars with relatively small diameter and length.

##### Cyclic Extrusion Compression

**Figure 3:**
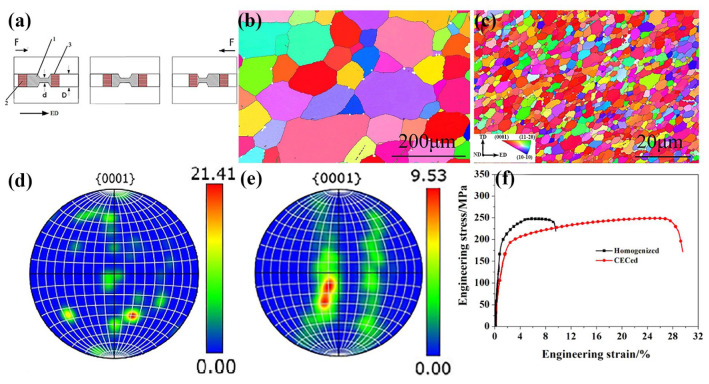
(**a**) The schematic sketch of CEC process [94]; (**b**–**e**) EBSD analysis of homogenized alloys and CEC-processed alloys: (**b**,**c**) IPF images, (**d**,**e**) (0001) pole figures; and (**f**) engineering stress–strain curves of the homogenized billet and CEC-processed alloys [91].

The cyclic extrusion–compression (CEC) technique was first conceptualized and developed by Richett, realizing the deformation by the reciprocating extrusion via an extruder, as shown in Figure 3a [92,94]. Due to its reciprocating extrusion, the CEC has a relatively high requirement for the plasticity of the biomedical Mg alloy. Compared with the conventional extrusion and ECAP, the main advantage of the CEC is its integration of multi-processes. It need not the repeating of filling billet in an extrusion mold, which ensures the consistency of the extrusion processing [93]. Secondly, the CEC could save much time and avoid the heating for multi-pass extrusions, which decreases the unnecessary procedure. Therefore, the biomedical Mg alloy could achieve the “knead dough effect”, eliminating the metallurgical defects effectively and achieving a homogenized, refined grain structure. In addition, the biomedical Mg alloy processed by CEC would possess uniform strain and stress distribution inside, which benefits the subsequent processing. Since these features, the CEC processing could well fragmentate the secondary phases and uniform their distribution, which contributes to the plasticity and corrosion resistance of the biomedical Mg alloy [93,145,146,147]. Because of the “knead dough effect”, the texture feature of the processed Mg alloy could be effectively weakened, contributing to its isotropic mechanical performance [55,57,58,103,107]. For instance, Sheng et al. [91] demonstrated that the application of CEC as a pre-processing for the Mg alloy billet could significantly enhance its processability and reduce the total processing cycle. With the application of the CEC, the grain and secondary phase in the ZE21B Mg alloy had been refined significantly (Figure 3b,c). Comparatively, the CEC processing had not increased the texture obviously, indicating its homogeneous deformation (Figure 3d,e). Such an optimized microstructure increased the elongation of the ZE21B alloy significantly, compared to the heat-treated alloy (Figure 3f).

During the CEC processing, the biomedical Mg alloy billet has to experience the extrusion force and the back-extrusion force, which requires the precision setting of the corresponding parameters. Specifically, the higher or lower forces would influence the deformation effect of the CEC processing, which also limits its rapid application for biomedical Mg alloys [92,148]. Therefore, the selection of appropriate CEC parameters plays a pivotal role in processing biomedical Mg alloys, implying the customization of parameters for specific alloys.

#### 2.1.2. Hot Rolling

Hot rolling is another material processing technique that is widely used for biomedical Mg alloys. It is a pressure processing method in which the alloy billet is deformed through the gap of a pair of rotating rolls (various shapes) [111,149,150]. During the hot rolling, the cross-sectional thickness of the Mg alloy billet is reduced, and the length is increased. Based on the deformation of materials, the type of hot rolling for biomedical Mg alloys could be classified into conventional rolling, cross-rolling, and cumulative rolling. The application of conventional rolling in the processing of biomedical Mg alloys enhances the mechanical properties by the microstructure regulation. Combined with a high deformation ratio and a certain temperature, the DRX would occur and influence the microstructure and mechanical performance obviously [150]. Taking full use of this process, the microstructure, secondary phase morphology, texture, and crystal defects could be well optimized, which helps the achieving of desired goals. Guan et al. [112] developed a biodegradable Mg-Zn-Sr alloy via conventional rolling, which demonstrated that the deformation produced by rolling refined the grain structure and secondary phases, achieving remarkable enhancements in mechanical properties and corrosion resistance. Actually, there are several parameters that influence the rolling effect on biomedical Mg alloys, including rolling temperature, rolling speed, reduction rate, and rolling passes [151,152,153,154]. The determination of the optimal rolling parameters for biomedical Mg alloys should be based on its initial plasticity, elevated temperature strength, secondary phase morphology, and so on [155].

##### Conventional Rolling

**Figure 4:**
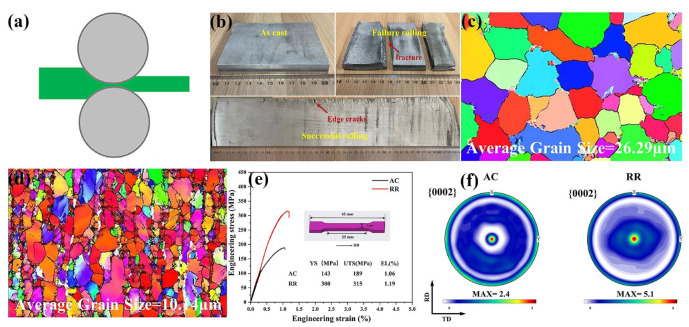
(**a**) Schematic diagram of the conventional rolling [95]; (**b**) Mg-RE alloy in different states; (**c**) EBSD results of as-cast (AC) alloys; (**d**) EBSD results of hot-rolled (HR) alloys; (**e**) mechanical properties of the AC and HR alloys; and (**f**) XRD results of AC and HR alloys: macrotexture [83].

Conventional rolling is a typical and simple rolling technique with a two-roller structure, and its advantages are simple craft, low cost, and wide adaption for metal processing, as shown in Figure 4a [151,152]. Research has shown that the volume fraction of coarse deformed grains in Mg alloys demonstrates an increasing tendency with increased rolling temperature. The excessively high rolling temperature causes obvious grain coarsening and texture, but the DRX is restrained, subsequently decreasing the age-hardening effect for the rolled biomedical Mg alloy [95,96]. Owing to the relatively low melting point, the rolling temperature should be lower, while the relative plasticity of Mg alloys requires a higher rolling temperature. To balance these requirements, the temperature for conventional rolling is generally set in the scope of 350~450 °C [152,156]. Actually, conventional rolling mainly constrains the deformation between rollers as there are two dimensions for free deformation, which would lead to edge cracking in the condition of excessive deformation [153]. Thus, the yield strength and ultimate tensile strength or compressive strength should be considered fully to obtain the rolled billet, benefiting the subsequent rolling. On the contrary, the insufficient rolling reduction fails to induce DRX and mainly induces crystal defects, resulting in suboptimal mechanical properties of the rolled Mg alloy [157]. The rolling reduction and temperature cooperate together to control the DRX in the rolled Mg alloy, which demands strict selection.

Deng et al. [83] investigated the Mg-RE alloy fabricated by conventional rolling with multi-passes at 500 °C and revealed that minimal cracking was generated in the rolled plate at the single reduction of 5%, suggesting that the accumulated small reduction at a relatively high temperature could realize good deformation in the Mg-RE alloy (Figure 4b). EBSD analyses revealed that such a rolling parameter could obtain the microstructure with a fine and uniform grain structure (Figure 4c,d). The rolling also induced an increase in texture intensity, by which the strength of the alloy enhanced greatly, but the ductility was also kept similar (Figure 4e,f). The texture effect has counteracted the ideal goal of the optimization of mechanical properties and further affected the subsequent processing. For this condition, the assisting treatment should be adopted to eliminate the texture and recover the anisotropy of rolled biomedical Mg alloys [158,159,160,161].

Based on the research, conventional rolling could realize the processing of biomedical Mg alloys by one-dimension constrained deformation and two-dimension free deformation. However, the approximate unidirectional rheological behavior during rolling leads to the high texture intensity in the rolled biomedical Mg alloy, decreasing the plasticity and harm to subsequent processing. Moreover, the isotropic mechanical properties would influence the service life of the fabricated implant because the failure happens in the weak orientation. Even though the heat treatment could mostly solve the problem, it also results in the grain coarsening and counteracts the grain refinement.

##### Cross-Rolling

**Figure 5:**
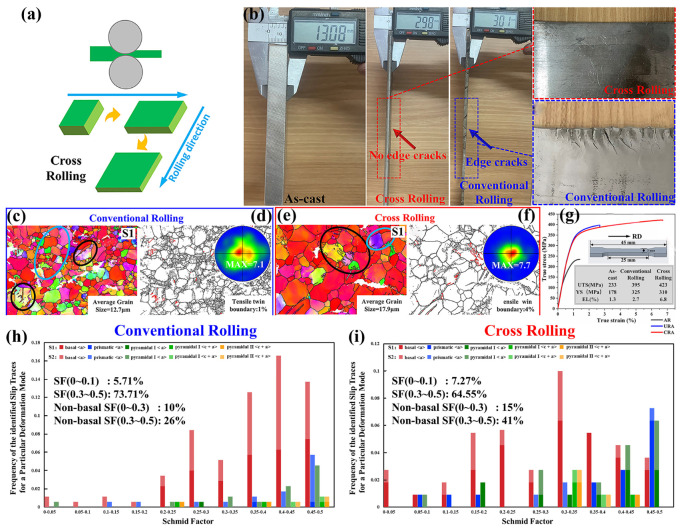
(**a**) Schematic diagram of cross-rolling; (**b**) the as-cast Mg-RE alloy and its sheet after cross-rolling, the RE-doped Mg alloy sheet after conventional rolling; (**c**–**f**) EBSD results of the conventional-rolled and cross-rolled specimens in the original stage (s1) in the in situ tensile test; (**g**) mechanical property test results of as-cast Mg-RE alloys and the sheet after conventional rolling and cross-rolling; and (**h**,**i**) frequency and Schmid factor distributions of activated slip systems at different stages of plastic deformation [95].

To address the problem that existed in conventional rolling, cross-rolling (CR) was developed, which changes the rolling direction to weaken the single-orientation texture [95]. Specifically, the first rolling direction has a 90° degree, and such an alternation continues in all rolling processing (Figure 5a) [95]. Extensive research demonstrated that cross-rolling significantly refines the grain and weakens the texture of the biomedical Mg alloys, alleviating anisotropy, which enhances mechanical performance and corrosion resistance [162,163,164,165,166,167,168]. Zhang et al. [168] successfully fabricated Mg-2Zn-2Gd alloy plates via CR followed by annealing treatment, achieving remarkably low anisotropy in mechanical properties between the rolling direction (RD) and the transverse direction (TD).

Similar to conventional rolling, the critical parameters of CR mainly include rolling temperature, rolling reduction, and rolling passes, which determine the microstructure and mechanical performance along different directions. Considering the feature of CR, the rolling temperature is a little higher, and the preferred values range from 400 °C to 500 °C, while the rolling reduction varies between 5% and 30%. The appropriate CR parameters could markedly enhance the overall performance of the biomedical Mg alloys [163,165,166,167,168]. In particular, the variation of rolling passes could exert an obvious influence on the texture and grain structure [167]. Ji et al. [98] demonstrated that the regulating of rolling reduction at the central and edge regions effectively reduced the edge crack depth and weakened basal texture at the edges, which helps the microstructure homogenization. However, this method may induce non-uniform deformation across the plate [95]. The subsequent annealing treatment could significantly reduce texture intensity and improve the anisotropy of Mg alloys [162,164,167,168]. Tian et al. [95] fabricated Mg-RE alloy plates via cross-rolling (Figure 5b) and observed a notable reduction in average grain size than that fabricated by conventional rolling, which just increased the basal texture slightly (Figure 5c–f). Compared with as-cast and conventionally rolled Mg-RE alloys, the CR enhances the mechanical properties significantly (Figure 5g). During the plastic deformation of the Mg-RE alloy induced by cross-rolling, the activation of non-basal slip systems was increased, which mainly controlled the deformation process (Figure 5h–i).

The CR improves the ductility and strength simultaneously by refining grains and the secondary phase, homogenizing stress distribution, and weakening texture, which benefits homogeneous corrosion or degradation [95,168]. The reasonable CR parameters and assisting treatment should be designed to obtain the ideal mechanical and corrosion performance.

##### Accumulative Roll Bonding

**Figure 6:**
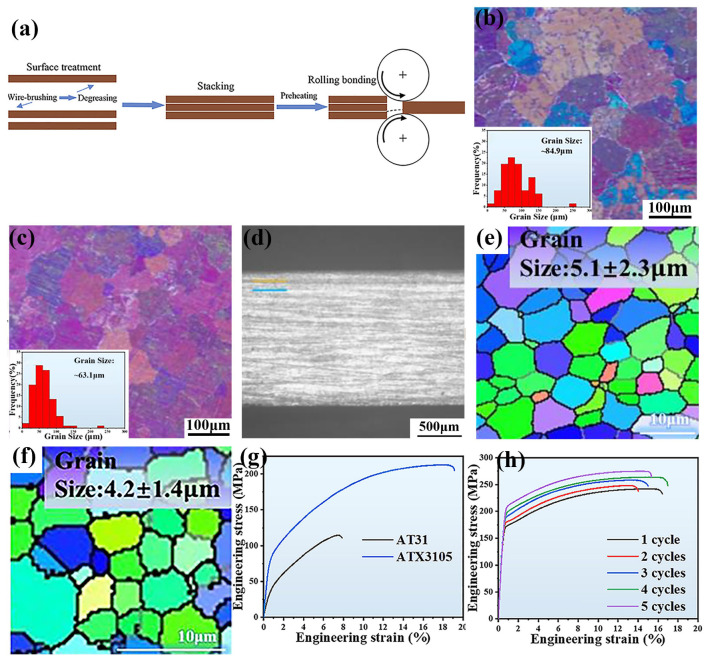
(**a**) Schematic diagram of accumulative roll bonding [153]; OM images of as-homogenized (**b**) AT31 and (**c**) ATX3105 alloys; (**d**) OM images of annealed ARB5 composite sheets; (**e**,**f**) high magnification IPF images and misorientation angle distribution of different regions in annealed ARB sheets: (**e**) AT31 layer ARB5, (**f**) ATX3105 layer ARB5. (**g**) Engineering stress–strain curves of as-homogenized AT31 and ATX3105 alloys at room temperature. (**h**) Engineering stress–strain curves of annealed composite sheets at room temperature along the rolling direction (RD) under different ARB cycle numbers [170].

Due to less activation of the slip system, the high deformation of biomedical Mg alloys is always a significant challenge. The accumulation of small deformation could achieve the final goal, but there would be great inner stress and texture, which decreases the isotropy of mechanical properties. The development of accumulative roll bonding (ARB) provides a method to improve the deformation behavior of the biomedical Mg alloy, whose procedure is the repeating of stacking of rolled plates, welding, and rolling, as shown in Figure 6a [153]. Then, the Mg alloy plate could be accumulatively deformed and cause great deformation on the initial plate. Benefiting from the ultra-high deformation, the ultra-fine grain size with a submicron size could be obtained, which increases the strength of the Mg alloy significantly [153,169]. Due to its features and advantages, the ARB is widely utilized to fabricate metal with ultra-fine grain size and a metal-based composite [170,171]. The ARB process typically involves a significant reduction in thickness to promote enhanced plastic deformation. This technique can be performed at elevated temperatures, and the ARB at high temperatures could achieve improved interlayer bonding, enhanced strength, and refined grain structure [99]. When it is conducted within a temperature range of 350 °C to 450 °C, the ARB processing effectively refines the grain structure of Mg alloys. Through multiple cycles of deformation and recrystallization, the grains are progressively refined and have a uniform distribution, accompanied by a high density of dislocations and structural defects, by which the strength, tensile strength, and hardness of the biomedical Mg alloy is increased significantly [100,101]. The former research revealed that the critical parameters of the ARB include the number of stacking cycles, rolling temperature, stacking speed, and the reduction ratio [100]. Additionally, the increasing of rolling passes enhances the interfacial bonding within Mg alloys or its composite, leading to improved structural integrity. Sun et al. [171] utilized the ARB to fabricate a Zn/Mg multilayer composite and demonstrated that the increased number of ARB cycles reduced the thickness of the Zn and Mg layers progressively, which introduced numerous heterogeneous interfaces. Li et al. [170] synthesized the AT31/ATX3105 Mg alloy composite using ARB, which obtained the average grain sizes of 84.9 μm and 63.1 μm for AT31 and ATX3105 layers, respectively (Figure 6b,c). With the increasing of rolling passes, the interface between the ATX3105 and AT31 layers progressively strengthens, demonstrating enhanced adhesion (Figure 6d). Additionally, following five ARB treatment cycles, the grain sizes of the AT31 and ATX3105 layers were substantially refined to approximately 5.1 μm and 4.2 μm, respectively (Figure 6e,f). After the fifth ARB cycle, the AT31/ATX3105 Mg alloy composite achieved optimal mechanical properties (Figure 6g,h).

Compared with the conventional rolling and CR, the ABR could realize the ultra-fine grain structure with a submicron size, which could improve the strength and ductility simultaneously by assisting with heat treatment. It was found that the ARB process can directionally change the crystal orientation and texture to obtain specific mechanical properties [100]. The detailed regulation of parameters helps the optimization of the comprehensive performance of the biomedical Mg alloy or its composite.

#### 2.1.3. Hot Forging

Forging is a widely applied manufacturing technique that applies compressive force through forging machinery to induce plastic deformation in metal billet, thereby producing components with specific mechanical properties and shapes [172]. Compared with hot extrusion and hot rolling, hot forging possesses special advantages, including a big processing dimension scope, high deformation force, flexible process, etc. Therefore, this effect could well eliminate casting defects in alloy ingots such as porosity, shrinkage, and shrinkage porosity. Due to its multi-directional deformation, the hot forging could well restrain the formation of strong texture, which benefits the strength and ductility synchronously [173]. Hot forging is frequently employed to achieve a more uniform microstructure and balanced mechanical properties [117,172]. Because of the relatively low high-temperature strength of the biomedical Mg alloys, the conventional hot forging techniques, such as hammer forging, die forging, and so on, are not suitable for their deformation. Especially for the Mg alloy with high alloying content, the conventional hot forging would lead to hot cracking and processing failure. The multi-directional and radial forging (spin forging) techniques are commonly used to improve the overall properties of biomedical Mg alloys. Martynenko et al. [117] investigated ZX11 Mg alloys subjected to spin forging (radial forging) and demonstrated that this technique significantly refined the grain structure and increased the strengths, maintaining a high elongation, which contributed to the well improved fatigue strength. In addition, it was observed that the spin forging had no adverse effect on the corrosion resistance of the ZX11 Mg alloy.

Generally, traditional hammer forging exhibits a non-ideal advantage for the hot deformation of Mg alloys, often leading to lower strength than extruded or rolled counterparts. Nevertheless, forging remains indispensable for the production of large components, whose advantages are suitable to be set as the initial processing [172]. Recent research predominantly emphasizes enhancing the properties of Mg alloys through advanced methods such as multi-directional forging, radial forging, die forging, and related techniques [174]. For the biomedical Mg alloy, its basic requirements in isotropic mechanical properties indicate the more suitable multi-directional forging and radial forging (rotary swaging) for this kind of metal [82]. In particular, the present investigation on hammer forging remains relatively scarce.

##### Radial Forging

**Figure 7:**
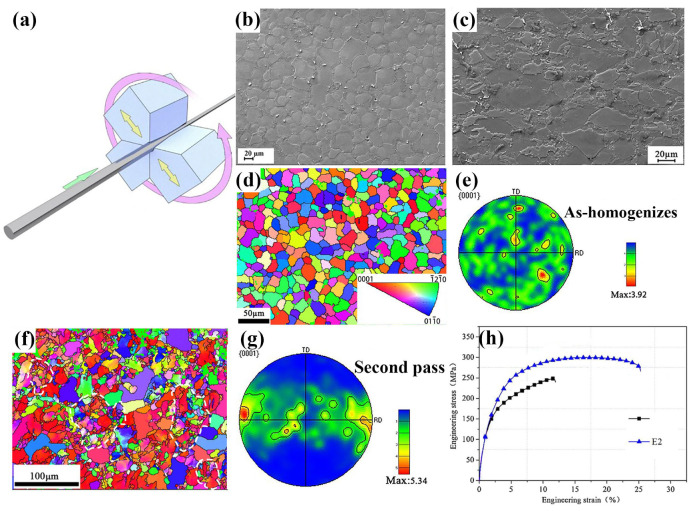
(**a**) Schematic diagram of radial forging [177]; (**b**) SEM patterns of the as-homogenized ZK60 Mg alloy; (**c**) microstructural evolutions of the ZK60 Mg alloy in edge areas after two passes of the RF process; (**d**–**g**) EBSD observation results of the ZK60 Mg alloy: (**d**,**e**) as-homogenized, (**f**,**g**) two passes of the RF process; and (**h**) tensile stress–strain curve of the sample after homogenization versus the edge of the second pass [82].

Radial forging (RF), also referred to as rotary swaging, is a low-cost-effective forging technique, which uses four radially arranged dies simultaneously to exert high-frequency radial impacts on the alloy workpiece [82]. Since the processing features, the RF is frequently applied to process the biomedical Mg alloy with a bar shape. The RF always involves some critical parameters, including the billet temperature, thinning rate, billet geometry, and inclination angle, as well as the feeding rate. Among these, billet temperature and feeding speed are the most critical ones. Typically, the billet temperature for RF ranges from room temperature to 400 °C [82,117,175]. Improper process parameter selection can readily lead to instability in the billet flow, adversely impacting the forging effect [175,176]. The previous research demonstrated that the RF effectively enhances the strength and ductility of Mg alloys by the refinement of grain structure, accompanied by slightly increased texture intensity [82,117]. Moreover, the alloy processed by RF exhibited unchanged corrosion resistance, which ensures its practical application in the processing of the biomedical Mg alloy [117]. Zuo et al. [82] fabricated a ZK60 Mg alloy with a typical bimodal grain structure (coarse grains with an average size of 14.1 μm and fine grains with an average size of 2.3 μm) using a two-pass RF technique (Figure 7a) [177]. The degree of grain refinement achieved via dynamic recrystallization (DRX) increases, and the twin boundaries disappear (Figure 7b,c). After two passes of radial forging, the ZK60 alloy exhibited a high fraction of DRX grains and a decreasing trend of average grain size (Figure 7d,f). The homogenized ZK60 alloy has a random texture. As the strain increases, the basal texture gradually weakens (Figure 7e,g). Furthermore, after two passes, YS and UTS increased to 180 MPa and 300 MPa, respectively, with an EL of 25.3%. It demonstrates its potential for high-performance applications (Figure 7h).

The RF can achieve a microstructure with a small size and fewer texture features, which is beneficial for the homogeneous performance in different crystallographic orientation. However, its efficiency is relatively low, and it requires stringent parameter control, which limits its widespread application. If used for the final processing of biomedical Mg alloys, its advantages could be fully taken and used.

##### Multi-Directional Forging

**Figure 8:**
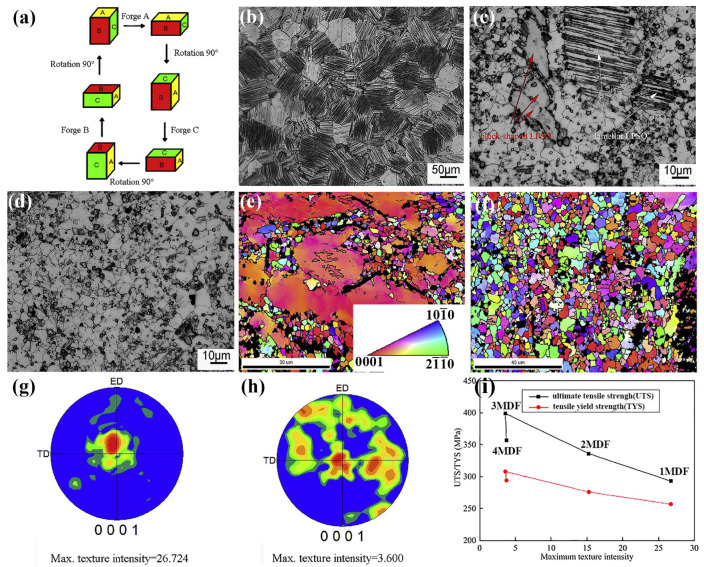
(**a**) Schematic diagram of multi-directional forging; (**b**) the OM micrographs of the as-homogenized Mg-13Gd-4Y-2Zn-0.5Zr alloy. (**c**,**d**) The OM images of the MDF-processed experimental alloy after (**c**) 1MDF: 480 °C, (d) 3MDF: 440 °C. (**e**,**f**) The inverse pole figure (IPF) map at a different MDF pass of the Mg-13Gd-4Y-2Zn-0.5Zr alloy: (**e**) 480 °C, 1MDF, (**f**) 440 °C, 3MDF. (**g**,**h**) Pole figures and maximum texture intensity of the samples with different MDF passes: (**g**) 1MDF: 480 °C, (**h**) 3MDF: 440 °C. (**i**) The relationship between maximum texture intensity and tensile properties of the MDF-processed alloy [178].

Compared with the RF, multi-directional forging (MDF) is a pivotal forging technique for the efficient fabrication of large-scale Mg alloy components with high performance (Figure 8a) [178]. Since no specific space limitation, the size of the forging ingot could be bigger, but its shape should be a cube, which benefits the synergistic deformation in three dimensions. The critical parameters of MDF are forging temperature, forging passes, and forging rate, which influence the quality of the forged alloy [178,179]. The research on Mg alloys processed by MDF demonstrated that the increases in forging temperature weakened the particle-stimulated nucleation (PSN) effect and decreased the formation of dynamic precipitates, while the promoted activation of the cone slip accelerated grain boundary migration, by which the continuous dynamic recrystallization (CDRX) progressively dominated deformation and caused significant microstructural evolution [179]. Within an optimal forging temperature range, the formation of dynamic precipitates can be effectively suppressed, and excessive grain growth can be mitigated [178,179]. Furthermore, the forging passes exert an important influence on the microstructure and mechanical properties of the biomedical Mg alloys. Under optimal forging pass conditions, the recrystallization rate of the alloy increases progressively with the rise in the initial forging temperature. When the initial forging temperature is defined, the increasing of forging passes leads to the gradual refinement of grains and reduction in maximum texture density, thereby significantly improving the alloy’s anisotropy and mechanical properties. However, the enhancement of mechanical properties is slight, when the forging pass exceeds a certain threshold [104].

Ramesh et al. [65] demonstrated that continuous MDF significantly refined the grain size of Mg alloys and produced a more uniform grain size distribution. The deformation resistance and tensile strength of the alloy, along with its corrosion rate, were improved. Wang et al. [180] observed twins and high-density dislocations in the alloy induced by MDF, which catalyzed the nucleation of the twinned variants, thereby further refining the grain structure. The synergistic interaction between dislocations and twins enhanced the strength and plasticity of the alloy. Dong et al. [178] reported that the average grain size of the homogenized Mg-13Gd-4Y-2Zn-0.5Zr alloy was significantly reduced to 4.0 μm after three cycles of MDF processing (Figure 8b–d). The area fraction of DRX grains increased to 97.3% after three forging cycles, indicating the almost completed DRX (Figure 8e,f). With the increase in forging cycles and cumulative strain, the texture gradually changed from a strong basal texture to a random distribution (Figure 8g,h). The alloy reached its peak ultimate tensile strength (UTS) and tensile yield strength (TYS) after three forging cycles, and decreased UTS and TYS were observed after four forging cycles (Figure 8i).

Conversely, Wang et al. [181] investigated the corrosion behavior of the ZK60 alloy after different MDF processing and revealed that excessive forging cycles can exert a negative impact on corrosion resistance. The corrosion resistance peaked after 8 MDF cycles and then declined significantly with further MDF processing even beyond 15 MDF cycles. This finding underscores the importance of selecting an optimal MDF cycle to maximize the corrosion resistance of the biomedical Mg alloy. Moreover, the pretreatment could contribute to enhancing the deformation capacity and overall properties of the biomedical Mg alloys. The experimental results indicated that low-temperature pretreatment prior to MDF obtained higher improvement in the deformation capability of Mg alloys, which increased mechanical properties further [182,183]. Compared with as-cast Mg alloys, the forging could improve their mechanical properties significantly, which helps the application of biomedical Mg alloys [184,185].

In general, the processing techniques of hot extrusion, hot rolling, and hot forging have a profound effect on the microstructure of biomedical Mg alloys, promoting grain refinement, secondary phase, and texture evolution, which could effectively improve mechanical properties and corrosion resistance. Due to the different rheological behaviors of biomedical Mg alloys in these processing techniques, the texture evolution exhibits different evolution tendencies, alongside the refined grain structure. The hot extrusion and hot rolling increase the texture intensity mainly along the single deformation direction, while the hot forging could restrain the increase in texture intensity, due to its multi-direction deformation feature [186,187,188]. Due to the close relation between microstructure and mechanical properties, the combination of different processing techniques sequentially would be a better choice for biomedical Mg alloys.

### 2.2. Mechanical Properties of Biomedical Mg Alloys

Because of the balanced requirements in mechanical properties, corrosion resistance, and cytocompatibility, biomedical Mg alloys should take full consideration of chemical composition, microstructure, and secondary phase [3]. It means that the specific alloy should fully meet the mechanical and biological requirements. As described above, the different alloying elements in biomedical alloys exert specific influences on certain performances. Therefore, the biomedical Mg alloys would have a different initial microstructure and mechanical properties in as-cast states, which indicates that their processing would be diversified as well. Furthermore, the detailed clinical application objectives also have specific demands in strength, ductility, toughness, corrosion biodegradation behavior, etc. As a result, the biomedical Mg alloys need specific processing to achieve the corresponding performance. However, from the mechanical performance perspective, the ideal biomedical Mg alloys must possess adequate tensile strength and ductility to ensure reliable durability and safety under complex loading conditions [1,10].

The research demonstrated that employing appropriate deformation techniques can markedly improve the mechanical properties of biomedical Mg alloys [66]. Consequently, the optimization of deformation processing has emerged as a main focus in this research field, to address the specific demands of biomedical applications. For instance, Kang et al. [172] studied the influence of casting and forging on the microstructure and mechanical properties of AM60 and AZ31B Mg alloys. Their findings revealed that casting processes tend to introduce defects, leading to reduced tensile strength and fatigue resistance. In contrast, the thermomechanical processing on these Mg alloys obviously demonstrated increased mechanical properties. Based on the described thermomechanical processing, the mechanical properties of a typical biomedical Mg alloy with a diversified alloying composition are summarized and given in Table 3.

Clearly, more biomedical Mg alloys have adopted the hot extrusion technique for processing, which indicates the convenience, adaptability, and effectiveness of this kind of technique. Even though the biomedical Mg alloys are processed by the same hot extrusion, their mechanical properties still differ obviously. The alloys with small alloying contents always have a relatively lower strength, but their elongations are relatively higher. The alloys with high alloying contents would possess higher strength after hot extrusion, but their elongations diversify differently. If the small RE is added to the alloy, the elongation could be increased obviously. Comparatively, the biomedical Mg alloys processed by hot rolling could help to increase strength, but the elongation is relatively low. The forging could increase the strength of the biomedical Mg alloy obviously, especially in yield strength, and keep the elongation at in acceptable level.

### 2.3. Biological and Corrosion Performance of Biomedical Mg Alloys

As a kind of metal for clinical implants, the biomedical Mg alloys should endure the in vivo environment and varied loading, and the specific implant shapes also impose more requirements. Nowadays, biomedical Mg alloys have been applied in clinical implants such as bone fixture plates and screws, cardiovascular stents, hemostatic clips, and so on [9]. Compared with the conventional implant prepared from steel or titanium alloys, the strength of biomedical Mg alloys is still not high enough, which determines the present developed implants to be mainly applied in positions with small loading. Though the greatest advantage of biomedical Mg alloys is biodegradation in an in vivo environment, its corrosion rate is still critical and required in a reasonable value [196,197,198]. Therefore, the in vitro and in vivo experiments are necessary evaluations to determine the safety and effectiveness of biomedical Mg alloys, by which the biodegradation behavior, bio-functions, and bio-toxicity could be investigated as well [122].

**Figure 9:**
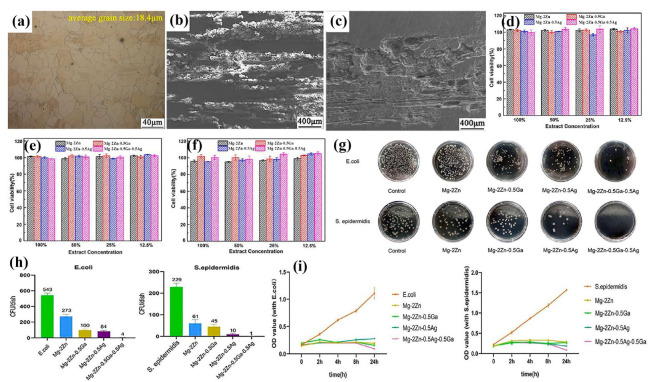
(**a**) Optical micrographs of as-extruded Mg-2Zn-Ga-Ag alloys. (**b**,**c**) Corrosion morphology of as-extruded Mg-2Zn-Ga-Ag alloys. (**d**–**f**) Viability of MC3T3-E1 cells after incubation in different extract concentrations of as-extruded Mg-2Zn-Ga-Ag alloys for 24 h, 48 h, and 72 h. (**g**) Images of colonies on Mg-2Zn-Ga-Ag alloys co-cultured with *E. coli* and *S. epidermidis*. (**h**) CFU of *E. coli* and *S. epidermidis* after incubation with Mg-2Zn-Ga-Ag alloys for 24 h. (**i**) OD values of *E. coli* and *S. epidermidis* after incubation with Mg-2Zn-Ga-Ag alloys for 24 h [198].

In general, in vitro experiments are a convenient and efficient method to reveal the basic biological properties of the biomedical Mg alloy. There are several kinds of simulated corrosion media frequently employed to replicate physiological conditions, which could systematically evaluate the biodegradation behavior of biomedical Mg alloys. The commonly utilized simulated corrosion media include simulated body fluids (SBFs), 0.9% sodium chloride (NaCl) solution, Hank’s balanced salt solution (HBSS), phosphate-buffered saline (PBS), and Dulbecco’s modified eagle’s medium (DMEM) [36,197]. These media are designed to replicate the chemical environment of the human body, thereby offering reliable tests for biomedical Mg alloys. Fan et al. [190] performed in vitro investigations involving the immersion test of an extruded Mg-1.5Y-1.2Zn-0.44Zr alloy in simulated body fluid (SBF). Their findings revealed that the alloy enhances osteoblast activity and exhibits excellent cytocompatibility. No significant cytotoxicity was observed in L-929 cells, and the alloy extract demonstrated a time-dependent promotion of cell proliferation [190]. Similarly, Wang et al. [198] investigated the corrosion behavior, cytotoxicity, and antimicrobial behavior of the extruded Mg-2Zn-Ga-Ag alloy with an equiaxed grain structure (Figure 9a). After 500 h of immersion in Hank’s solution, the alloy surface was covered with a continuous corrosion film and porous corrosion products (Figure 9b). The deep corrosion cavities could be observed on the surface after removing corrosion products, indicating mixed corrosion behavior with intensified localized corrosion (Figure 9c). Cytotoxicity assays demonstrated that the alloy exhibited no toxicity toward MC3T3-E1 cells (Figure 9d–f). Due to the presence of Ag in the alloy, it also displayed a remarkable antibacterial effect (Figure 9g–i).

Actually, the in vivo experiments would provide the accurate and reliable reports, due to the relatively similar body environment. For different clinical implants, there are different animal models for biomedical Mg alloys, including rats, beagle dogs, New Zealand rabbits, and pigs, which provide specific tissue defects. The in vivo experiments help to systematically investigate the interactions of biomedical Mg alloys with tissues in complex physiological environments, which comprise the assessment of inflammation, metabolism, histotoxicity, bioactivity, etc. Long-term dynamic monitoring facilitates a comprehensive understanding of their degradation behavior under practical application conditions and its impact on tissue healing and functional recovery, thereby providing robust evidence for their clinical implementation [197,199]. However, the in vivo experiment is an expensive test, which consumes much time and funds. In comparison, the in vitro tests are more suitable for the initial selection of biomedical Mg alloys, especially for the corrosion rate test. The corrosion rates of a typical biomedical Mg alloy prepared by different thermomechanical processing are listed in Table 4.

From these data, it can be found that the alloying elements and processing technique both exert an obvious influence on the corrosion behavior. For the biomedical Mg alloy prepared by hot extrusion, the decreasing of alloying content is beneficial to decrease the corrosion rate. It is interesting that the addition of more RE in the biomedical Mg alloy always results in a higher corrosion rate, which might be attributed to the formation bulk secondary phase. For the biomedical Mg alloy with similar low alloying content, the hot rolling could obtain a lower corrosion rate, which may be ascribed to an ultra-fine grain structure. Comparatively, the biomedical Mg alloy processed by hot forging demonstrates a moderate corrosion rate.

## 3. Applications of Biomedical Mg Alloy

With the help of thermomechanical processing, the mechanical properties, corrosion resistance, and cytocompatibility of biomedical Mg alloys have been well improved, which promotes their clinical application as implants or devices. Considering the non-ideal strength of the biomedical Mg alloys, they are mainly applied in the orthopedic, cardiovascular, dentistry, gastroenterology, and oncology fields with small requirements in mechanical properties. Currently, the implants fabricated from the biomedical Mg alloys comprise hemostatic clips, intramedullary nails, bone nails, bone plates, cardiovascular stents, guided bone regeneration (GBR) membranes, biliary stents, etc. The successful implementation of biomedical Mg alloys in these clinical fields not only extends their application exploration but also reveals critical insights into this kind of novel biodegradable alloys.

### 3.1. Hemostatic Clip

A hemostatic clip is frequently utilized in minimally invasive surgery to realize the rapid occluding of blood vessels, achieve effective hemostasis, and regulate blood flow direction, which ensures the successful completion of surgery [49]. Previously, the hemostatic clips were predominantly fabricated from titanium, particularly U-shaped titanium variants, whose excellent deformability and strength help to achieve vascular occlusion immediately. However, as an inert metal, the titanium hemostatic clip has to stay in the tissues permanently post-surgery or be taken out by secondary surgery. If the titanium hemostatic clip stays in the body as a foreign object, it would induce potential foreign-body reactions, including inflammation, pain, and the formation of calculi [208]. Moreover, the prolonged existence of titanium hemostatic clips can result in metal artifacts in CT imaging, compromising the quality of diagnostic images, while, in magnetic resonance imaging (MRI), they may impair the accuracy of image acquisition. The surgical removal of titanium hemostatic clips poses a potential risk of inflicting additional harm to the patient [209,210].

To address these problems, researchers have proposed that the next generation of hemostatic clips should possess biodegradable properties, allowing for gradual degradation after achieving hemostasis and tissue repair, with degradation products safely absorbed by the human body [50]. Biomedical Mg alloys have emerged as promising alternative materials, owing to their exceptional cytocompatibility and biodegradability. Actually, the strength and plastic deformability of pure Mg could not fully meet the requirement of hemostatic clips. Consequently, biomedical Mg alloys with optimized mechanical properties and corrosion resistance have been applied to develop novel degradable hemostatic clips [211]. Regarding the function of hemostatic clips, the used biomedical Mg alloy must retain adequate mechanical strength for a minimum of two weeks after its clipping [49,50]. Moreover, the hemostatic clip prepared by biomedical Mg alloy has demonstrated significantly reduced metal artifacts in CT imaging compared to titanium hemostatic clips, with no notable complications observed postoperatively [209,210,211,212].

**Figure 10:**
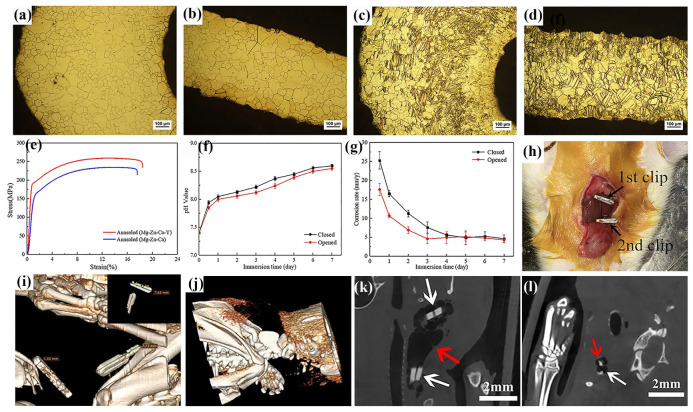
(**a**–**d**) The microstructure of the clip in its (**a**,**b**) opened and (**c**,**d**) closed states. (**e**) Stress–strain curves of annealed Mg-Zn-Ca-Y and Mg-Zn-Ca alloys. (**f**) The pH value of SBF during the immersion of the Mg-Zn-Ca-Y alloy clip. (**g**) The corrosion rate of the Mg-Zn-Ca-Y alloy clip. (**h**) The clip occludes carotid blood vessels of rats and cuts the blood vessel from the middle of the two hemostatic clips. (**i**,**j**) Micro-CT 3D images of Mg-Zn-Ca-Y hemostatic clips after different times of implantation: (**i**) 0.5 months and (**j**) 8 months. (**k**,**l**) Micro-CT cross-sectional images of the Mg-Zn-Ca-Y hemostatic clip after different times of implantation (white arrows point to the hemostatic clip, red arrows point to hydrogen): (**k**) 0.5 months and (**l**) 3 months [49].

Further investigations have confirmed the safety and promising application potential of biomedical Mg alloy hemostatic clips. For instance, the hemostatic clips prepared from extruded Mg-Zn-Ca alloys exhibited excellent mechanical stability and cytocompatibility in animal studies and retained effective clamping performance for two weeks, thereby fulfilling the clinical requirements for hemostatic clips [50,212]. In both mouse vascular closure tests and rabbit fallopian tube occlusion tests, no abscesses, tissue necrosis, or significant adverse effects were observed, and the wounds exhibited normal healing throughout the observation period [212]. Yu et al. [49] fabricated V-shaped hemostatic clips prepared by a Mg-3Zn-0.2Ca-0.5Y alloy and observed the significantly increased twins and enhanced local hardness after the clipping deformation (Figure 10a–d). The biomedical alloy demonstrated high strength, elongation, exceptional mechanical properties, and corrosion resistance (Figure 10e–g). In rat experiments (Figure 10h), the hemostatic clips prepared from the Mg-3Zn-0.2Ca-0.5Y alloy were fully biodegraded within 8 months postoperatively, with no adverse tissue reactions observed (Figure 10i,j). Additionally, the hydrogen bubbles produced during the degradation of the hemostatic clips were markedly diminished in the later stages, and a small amount of gas was excreted via body fluids without leaving any residue (Figure 10k,l). Zhang et al. [213] demonstrated that P-type hemostatic clips fabricated from the Mg-Zn-Nd-Zr alloy achieved uniform vessel closure without gap formation, effectively mitigating stress concentration, and maintained secure clamping for seven days. Some investigations have studied the influence of various structural designs, such as transverse and R-shaped staggered hemostatic clips, on performance, which demonstrated excellent mechanical stability with no evidence of cracking following clamping deformation [214,215].

For the hemostatic clip, good deformability is a fundamental requirement, while strength is another important property. During the stitching process, the biomedical Mg alloy must ensure that no cracks are generated. Due to its relatively short service life, corrosion resistance is not the primary consideration. Therefore, a biomedical Mg alloy with high strength and ductility would be more suitable for this type of implant.

### 3.2. Bone Screw

In orthopedic surgery, the bone screw is widely used for the fixture of fractured bones, assisting tissue healing [216,217]. Currently, the main bone screws are mainly made from stainless steel and titanium or its alloys. These conventional metals could well realize the fixture, but they also have some disadvantages, such as toxic ions releasing, stress concentration, and so on, which result in secondary surgery for removal and thereby additional risks to patients [3]. The emergence of biomedical Mg alloys has brought some innovative developments in this kind of clinical implant [216,217,218,219]. The feature of biodegradation could eliminate the secondary surgery, and its elastic modulus closing to the human bone would eliminate “the stress shielding” effect [216]. Moreover, the density of biomedical Mg alloys is close to that of human bone, minimizing the sensation of foreign bodies [9]. The most important is the degradation products of biomedical Mg alloys, which could have some bio-functions, such as osteogenesis induction, anti-inflammatory, antibacterial effect, and so on [218,219].

Generally, the cytocompatibility and effectiveness of the bone screw fabricated from biomedical Mg alloys had been well investigated and verified in previous research. Sefa et al. [216] fabricated three bone screws with different biomedical Mg alloys (Mg-10Gd, Mg-4Y-3RE, and Mg-2Ag) with extrusion deformation and conducted long-term implantation trials (6 and 9 months) in rabbit femurs. Their findings revealed that all three Mg alloy screws displayed comparable long-term osteogenic responses, underscoring their potential utility in bone repair applications. Bazhenov et al. [66] fabricated Mg-Zn-Ga-(Y) alloys via hot extrusion and systematically assessed their suitability as bone screws, which demonstrated their lack of cytotoxicity toward MG63 cells and satisfied corrosion resistance for orthopedic applications. Torkian et al. [195] fabricated the WE43 Mg alloy using multi-pass ECAP and additional extrusion processes, obtaining superior mechanical strength and corrosion resistance, which demonstrated high potential application as a hollow screw.

**Figure 11:**
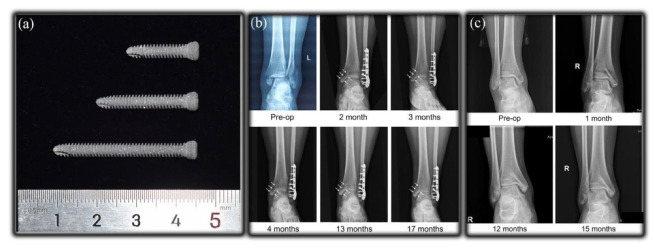
(**a**) The biodegradable Mg screw. (**b**) Preoperative and postoperative radiographs of a young female patient with a trimalleolar fracture. Two biodegradable JDBM screws (white arrows) are used to fix the medial malleolar fracture. (**c**) Preoperative and postoperative radiographs of a mid-age female patient with a medial malleolar fracture [217].

Clinical trials have further substantiated the potential of the biomedical Mg alloys prepared by thermomechanical processing as bone screw materials. For instance, the JDBM biomedical Mg alloy, developed by Prof. Yuan’s team at Shanghai Jiao Tong University in collaboration with Shanghai Innovative Medical Technology Co., Ltd. (Shanghai, China), has undergone clinical evaluation, demonstrating its safety and efficacy in treating medial malleolar fractures [217]. The research demonstrated that the coated bone screw prepared from extruded JDBM alloys is highly effective in promoting fracture healing, which alleviated postoperative pain and promoted joint function recovery by the degradation of the bone screw and ions releasing. Notably, no complications such as infection or fixation failure were observed, which verified the effectiveness of this bone screw in clinical application (Figure 11). Lee et al. [220] utilized a bioresorbable Mg bone screw (Resomet, U&I Corp., Seoul, Republic of Korea) to treat a Mason type II radial head fracture, demonstrating satisfactory outcomes in both imaging and clinical evaluations. Lam et al. [221] documented a clinical case involving three patients with elbow fractures, where Mg bone screws were employed to treat one radial head fracture and two humeral head fractures. Complete fracture healing was observed within three months post-surgery, with all patients regaining near-normal motor function. No complications, such as fixation failure, malunion, or infection, were reported, and the Mg bone screws degraded gradually. These clinical findings underscore the reliability and safety of using bone screws made from biomedical Mg alloys in orthopedic surgeries.

Actually, the bone screw is one of the hottest research topics for biomedical Mg alloys. Especially for orthopedic implants applied for small load bone fractures, the biomedical Mg alloy has many advantages. In 2015, the K-MET bone screws, made from Mg-Ca alloys and produced by U&I, were approved by the Korea Food and Drug Administration (KFDA), making them the world’s second Mg-based orthopedic implant device [222]. In China, the National Medical Products Administration (NAPA) approved the clinical trials of a degradable Mg bone fixation screw developed by Yi’an Technology in 2019. In addition, it obtained the certification of CE in 2020. In 2023, Yi’an Technology announced the completion of clinical trials for the biodegradable pure Mg bone nail, which was a significant advancement for medical Mg alloy application [223].

### 3.3. Bone Plate

Similar to bone screws, the bone plate is also a kind of implant widely used in orthopedic surgery, which fixes and stabilizes the fractured bone, providing basic support for the human body during bone healing [51]. The common surgery for bone plate fixation includes open reduction, internal fixation, and bridging [224]. Incisional reduction and internal fixation could improve the high accuracy of fracture alignment through anatomical repositioning, but they are prone to cause soft tissue damage and vascular disruption, prolonging recovery. In contrast, bridging fixation minimizes soft tissue damage but might increase the risk of fracture deformity and localized soft tissue compression. The primary function of a bone plate is to provide essential mechanical stability for the fracture site, particularly for weight-bearing bones like the tibia and femur, which raises strict requirements on the mechanical properties of bone plates [225]. Moreover, the bone plate plays an important role in fracture healing by regulating the biomechanical microenvironment along the fracture site, which requires the bone plate to have good elastic adaptation [51,224,225,226].

**Figure 12:**
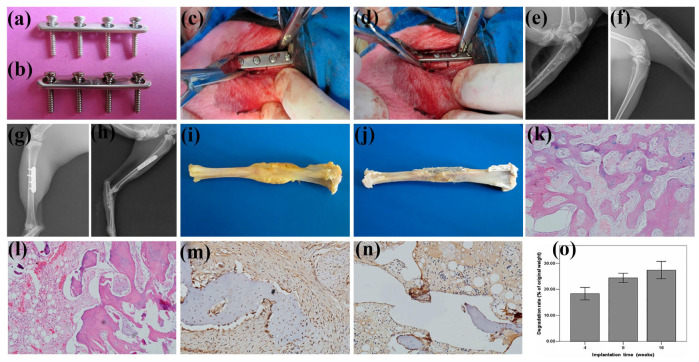
Photographs of (**a**) the Mg alloy plate and (**b**) titanium alloy plate. (**c**,**d**) Surgical procedure of the animal experiments. (**e**–**h**) X-ray photographs of the (**e**,**f**) Mg alloy plate and (**g**,**h**) titanium alloy plate implanted into the New Zealand white rabbit tibias at 4 and 8 weeks after surgery. (**i**,**j**) Photographs of the callus around the plates at 4 weeks post-implantation in the (**i**) Mg alloy plate group and the (**j**) titanium alloy plate group. (**k**,**l**) Pathologic photographs of the callus around the plates at 4 weeks post-implantation in the (**k**) Mg alloy plate group and (**l**) titanium alloy plate group (HE staining, ×100). (**m**,**n**) The expressions of BMP-2 in the new bone tissue around the plates at 4 weeks post-implantation in the (**m**) Mg alloy plate group and (**n**) titanium alloy plate group. (**o**) Degradation rate of the Mg alloy plate after 4, 8, and 16 weeks post-implantation [226].

As the bone screw, the traditional bone plate is mainly fabricated by stainless steel and titanium alloy, which could provide sufficient support but has some disadvantages, such as a high difference in elastic modulus and secondary surgery for removal [225]. The biomedical Mg alloy fabricated by thermomechanical processing could solve the problems above and provide relatively acceptable mechanical strength, which means it could potentially develop as an innovative bone plate [51]. In addition, the biomedical Mg alloy also needs to maintain adequate strength in 6~12 months for supporting bone healing and fully degrading within the subsequent 12 to 24 months. Then, its degradation rate should not exceed 0.5 mm/year to guarantee clinical safety. Wang et al. [226] investigated the effectiveness of biomedical Mg alloy bone plates in repairing tibial fracture by rabbit experiments (Figure 12a–d). Compared with the titanium alloy, the biomedical Mg alloy bone plate group exhibited a faster fracture healing rate and greater callus formation (Figure 12e–j). Callus formation (Figure 12k,l) and BMP-2 expression (Figure 12m,n) were significantly elevated. The in vivo observations indicated that the degradation of biomedical Mg alloy bone plates progressively rose with extended implantation duration (Figure 12o). These findings suggest that bone plates prepared from biomedical Mg alloys can effectively enhance callus formation and accelerate the osteogenesis stage. Gungor et al. [60] investigated the Mg-Zn-Ca-Mn alloy, highlighting its obvious advantages in preparing biodegradable bone plates. The alloy processed by hot rolling had a smaller corrosion rate than the standard degradation threshold (<0.5 mm/year) and superior mechanical properties compared to cortical bone, further validating its suitability for orthopedic applications. Rich et al. [227] designed a Mg0.45Ca alloy bone plate from a hot extruded alloy and treated its surface by plasma electrolytic oxidation (PEO). In experiments involving the fixation of osteotomy models on sheep zygomatic bones, the PEO coating effectively prevented an initial pH increase, lowering the pH at the fracture site. The bidirectional strain testing revealed that bone plate placement significantly influenced strain distribution in vivo, which might cause early failure. Rendenbach et al. [228] studied the WE43 Mg bone plate and revealed that the PEO surface modification significantly improved osseointegration and reduced the degradation rate during the initial 6 months post-implantation. These studies indicate that surface modification would be the best choice for the implants prepared from the biomedical Mg alloy, which regulates the microenvironment and benefits bone healing.

In clinical surgeries, orthopedic implants have the maximum usage, due to the largest group of patients. The MAGNEZIX^®^ CS, developed by Syntellix AG in Germany, is the world’s first biodegradable Mg alloy bone screw approved for implantation. It obtained the certification of conformity European (CE) in 2015 and received marketing authorization in Singapore in 2019 [229]. The application of biomedical Mg alloys in orthopedic implants has great influence, which encourages the following researchers. However, the relatively low strength of the biomedical Mg alloy is still a big challenge, which results in its application mainly in low-stress implants. The high-strength biomedical Mg alloy with a low degradation rate and good cytocompatibility would be the main research topic, which determines its extending application in the future.

### 3.4. Intramedullary Nail

The intramedullary (IM) fixation exhibits substantial advantages in achieving rigid fracture stabilization. It is extensively utilized in the treatment of long bone fractures, delivering robust stability and facilitating early weight bearing [230]. Compared to plate osteografting (PO), IM fixation exhibits superior biomechanical stability and reduced the nonunion rate significantly [230,231,232]. The related research has indicated that the IM fixture could decrease the disruption of the soft tissues around fractures. Moreover, it provides effective stabilization for simple diaphyseal fractures, which facilitates early joint mobility and mitigates stress concentrations at the fracture site [233]. Because of its minimally invasive feature, IM fixation could reduce approach-related complications, including soft tissue injury, hemorrhage, and postoperative infection, though it may elevate the risk of fat embolism [231]. Consequently, IM fixation surgery exhibits obvious advantages for patients with chronic comorbidities, such as diabetes mellitus, neuropathy, or peripheral vascular disease, which could decrease the risk of wound complications [51,234,235,236].

**Figure 13:**
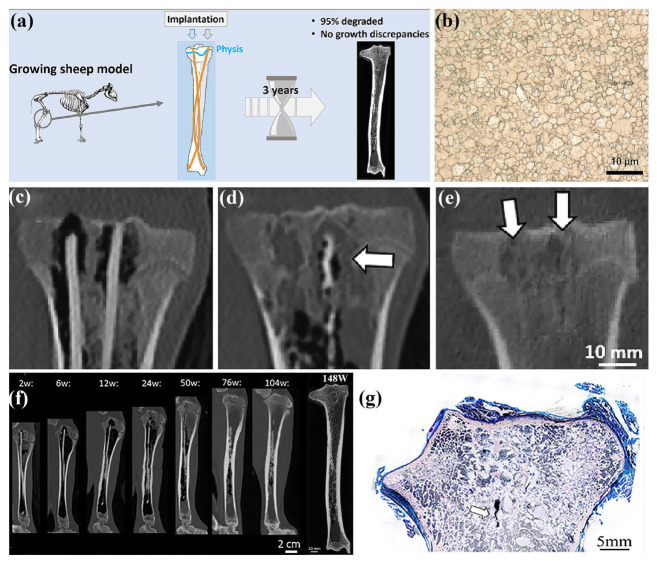
(**a**) Mg-Zn-Ca elastic stable intramedullary nail (ESIN) implantation across the growth plate does not affect longitudinal bone growth in juvenile sheep. Nearly 95% of the Mg-Zn-Ca ESIN material degraded within three years. (**b**) Microstructure of ZX00. (**c**–**e**) In vivo cCT images of ZX00 are shown for the time points (**c**) 2, (**d**) 50, and (**e**) 148 weeks after implantation; (**f**) In vivo cCT and ex vivo μCT images of ZX00-implanted animals, with in vivo cCT images of ZX00-implanted animal showing until 108 weeks post-implantation, ex vivo μCT data showing only a small radiolucent area around the ZX00 ESIN after 148 weeks. (**g**) Histological evaluation of tissue for a sheep treated with a ZX00 implant and euthanized after 148 weeks [202].

Currently, the intramedullary nail (IMN) is mainly fabricated by stainless steel or titanium alloy, which always requires secondary surgery for removal. Moreover, the IM position also benefits the application of biomedical Mg alloys, because its degradation products could upregulate calcitonin gene-related protein (CGRP) expression, which promotes the formation of new bone and blood vessels, accelerating traction osteogenesis [237]. The utilization of biomedical Mg alloys in the IMN has been investigated, which attempted to validate their effectiveness by involving diverse animal models [232,236]. Zheng et al. [238] demonstrated that Mg-containing mixed IMNs facilitated fracture healing in pretreated rats by enhancing CGRP synthesis and release. Sun et al. [239] observed that Mg implants significantly enhanced the bone tissue-to-volume ratio, which provides an effective approach for the dynamic monitoring of bone healing. Adam et al. [240] studied the degradation behavior of the Mg-1Ca alloy IMN in rabbit tibia and revealed no adverse effects on bone formation. Moreover, no pathological changes or gas embolism caused by corrosion products were observed in vital organs. Chow et al. [241] demonstrated that the Mg alloy IMN significantly increased the formation of new bone and maintained the required mechanical properties during its implantation in the rabbit patella fracture model. Yanagisawa et al. [48] revealed that the degradation rate of the IMN prepared from the biomedical Mg alloy was influenced by their shape and the surrounding tissue environment. Furthermore, the formed calcium phosphate layer on their surface promoted bone healing. Marek et al. [202] investigated the long-term in vivo degradation of the IMN prepared from the extruded Mg-Zn-Ca alloy in a sheep model (Figure 13a) and assessed their impact on the growth and development of bone. The extruded Mg-Zn-Ca alloy exhibited an average grain size of 1.87 μm, which contributed to its superior strength (Figure 13b). The final results exhibited that the Mg-Zn-Ca alloy IMN was almost fully degraded after the implantation of 148 weeks, and no adverse effects on bone growth or axial deviation were observed, which indicated that the Mg-Zn-Ca alloy with an ultra-fine grain structure and strength could be suitable for the IMN (Figure 13c–g).

In 2022, the biomedical Mg alloy hollow nail developed by Zhuoqia Medical has conducted clinical trials. The hollow nail, fabricated by the proprietary ZHUOMAG^®^ biomedical Mg alloy, was precisely designed with a thin wall structure, which imposes strict requirements on the degradation rate and mechanical properties [242]. The preliminary tests exhibited the effectively stabilized fractures by the hollow nail, and the degradation was small, by which no significant complications were observed postoperatively. As a kind of implant to fix the fractured long bone, strength is the primary requirement, which provides enough support to endure the external loading. The balanced corrosion rate is another requirement, which ensures long-term service life till the bone heals. The aforementioned animal studies have substantiated the potential applicability of biomedical Mg alloys in IMN applications, especially for the Mg alloy with an ultra-high strength and homogeneous ultra-fine grain structure. Nevertheless, the clinical safety and therapeutic efficacy of Mg alloys necessitate further rigorous investigation and validation to facilitate their broader adoption in the treatment of long bone fractures [243,244].

### 3.5. Cardiovascular Stent

**Figure 14:**
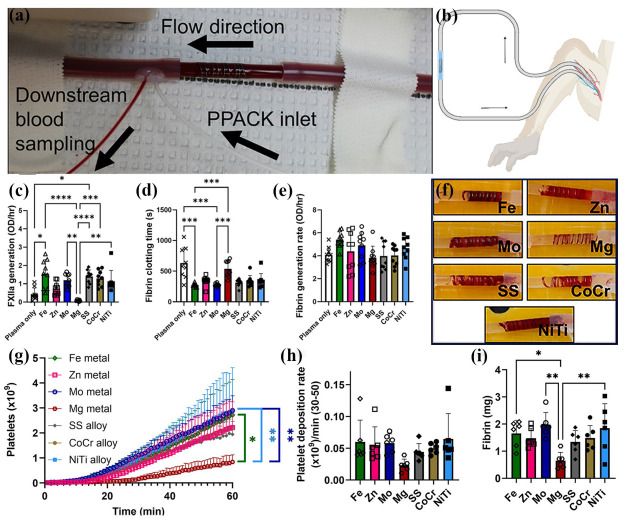
(**a**,**b**) Ex vivo whole blood testing on metal coils. (**c**–**e**) In vitro analysis of human platelet-poor plasma on metal wires compared to platelet-poor plasma alone for FXIIa generation and fibrin generation assays. (**f**–**i**) Ex vivo whole blood testing: (**f**) Representative images of each metal coil after exposure to flowing whole blood for 1hr at 100 mL/min. (**g**) Platelet attachment to metal wires, (**h**) the rate of platelet attachment from 30 to 50 min, and (**i**) fibrin on pure and alloyed metal coils after 1 h exposure to flowing whole blood without anticoagulants. (* indicates *p* ≤ 0.05. ** indicates *p* ≤ 0.01. *** indicates *p* ≤ 0.001. **** indicates *p* ≤ 0.0001) [247].

Different from implants for hard tissue repair, the cardiovascular stent demands strength and flexibility simultaneously, which indicates the diversified requirements in yield strength and ultimate tensile strength. The widely applied cardiovascular stents are mainly fabricated from stainless steel and cobalt base alloys, which could provide sufficient support for symptomatic vascular events. However, their permanent residence in the human body would cause a late thrombus problem, imposing an ultra-high risk on patients. Therefore, it is expected to develop the biodegradable cardiovascular stent, solving the present problems. The biomedical Mg alloy has been thought of as the most suitable metal for the cardiovascular stent, due to its attractive properties. Actually, the advancements in the processing of biomedical Mg alloys have enhanced their mechanical properties, providing some choices for biodegradable cardiovascular stents. As the implant for cardiovascular treatment, it has to face the interaction with vascular smooth muscle and the endovascular cortex, in which the possible inflammatory reaction would lead to endotheliosis and restenosis. Especially for the restenosis caused by the implanted Mg alloy, the stent remains a critical barrier for clinical application, primarily driven by endothelial hyperplasia and coagulation [245,246]. To assess the hemocompatibility of Mg, much research has been performed. Anderson et al. [247] studied four kinds of biodegradable metals (Fe, Zn, Mg, and Mo) alongside three widely utilized cardiovascular device alloys (NiTi, CoCr, and stainless steel). The in vitro hemocompatibility assays and non-human primate arteriovenous shunt model experiments provided further validation of the superior performance of pure Mg (Figure 14a,b). The results demonstrated that Mg markedly reduced platelet attachment, fibrin deposition, and FXII activation levels (Figure 14c–e). FXII (Factor XII) is the proenzyme of the plasma protease FXIIa, which initiates the plasma contact activation pathway, facilitates the generation of thrombin and bradykinin, and plays a critical role in thrombosis. Moreover, FXII is also involved in the inflammatory response. In flowing whole-blood experiments, the Mg still exhibited significantly lower platelet attachment compared to Mo, NiTi, and Fe, though it still displayed a certain degree of coagulation formation (Figure 14f–i). Consequently, the improved hemocompatibility of biomedical Mg alloys would be the key factor in promoting their clinical applications.

Presently, the main weakness of the biomedical Mg alloy compared to stainless steel or the CoCr alloy is its low strength. Based on the strategies of alloying, thermomechanical processing, and surface modification, the comprehensive performance of biomedical Mg alloys has improved significantly. The research has demonstrated that the cardiovascular stents prepared from biomedical Mg alloys provide enough radial support, acceptable vascular compliance, and lower acute retraction rates, by which no post-implantation cardiac death or stent thrombosis is reported [248]. In addition, Mg^2+^ ions released during Mg degradation have potential therapeutic value in controlling acute myocardial infarction and preventing atherosclerosis [249,250]. Since these features and developments, more biomedical Mg alloys were designed and studied for cardiovascular stents, including ZE21B [251], WE43 [252], and AZ31B [253]. These biomedical Mg alloys with specific processing demonstrate exceptional mechanical properties and corrosion resistance, making them highly suitable for developing biodegradable cardiovascular stents. For instance, the Mg-2Zn-0.46Y-0.5Nd alloy, produced via hot extrusion, has exhibited outstanding performance advantages for biodegradable stent applications [186]. Cai et al. [121] have developed the Mg-2Zn-0.6Zr-0.6Nd alloy with superior TYS, UTS, and EL, compared with other Mg alloys. Additionally, the application of functional surface coatings on biomedical Mg alloys markedly enhances their hemocompatibility and pro-endothelialization capabilities, further broadening their potential in cardiovascular applications [58,247,251,252,253,254,255,256,257,258,259]. Furthermore, the polydopamine (PDA)-bound fucoidan and collagen IV coating on the ZE21B alloy [251], the SBMA-AAM hydrogel coating applied to the AZ31B alloy [260], and the zinc oxide-loaded stearic acid coating on the WE43 alloy [261] all demonstrated excellent corrosion resistance and effectively promoted endothelial cell proliferation.

Benefiting from the abundant research, promising progress has been made in the commercialization of cardiovascular stents prepared from the biomedical Mg alloy [262,263,264,265]. Magmaris^®^, the first commercially available biodegradable Mg alloy stent, received the EU CE mark in 2016. As the first bioresorbable, drug-eluting metal stent, Magmaris^®^ has been deployed clinically in over 50 countries for treating stable coronary artery disease [262,263]. Clinical data indicate that Magmaris^®^ exhibits excellent radial support, enhanced vascular compliance, and a reduced acute retraction rate, with no reports of cardiac death or stent thrombosis [52]. However, late lumen loss within the stent persists as a significant challenge to the broader application of Mg alloy stents [263]. To address these challenges, Biotronik introduced an advanced iteration of the Magmaris^®^ stent with the launch of the next-generation absorbable stent, DREAMS 3G, in 2022. Constructed from a proprietary biodegradable Mg alloy, DREAMS 3G features a thinner yet stronger support framework and offers a range of sizes to suit diverse clinical needs. Recent studies revealed notable benefits in the formation of a stable calcium phosphate layer during stent degradation, contributing to prolonged support duration and a more consistent degradation rate [265]. Findings from the first-in-human study indicate that DREAMS 3G achieves enhanced safety and efficacy. It demonstrates the potential to address the limitations of traditional stents, offering greater radial strength, thinner stent profiles, and extended support duration [264].

### 3.6. Oral Implant

The recent studies on biomedical Mg alloys for applications in dentistry mainly focus on the guided bone regeneration (GBR) membrane, coating for dental implants, dentoalveolar fixation screws, and soft tissue regeneration [266]. The GBR is a basic technique in the therapeutic management of alveolar bone defects, which employs a barrier membrane to segregate gingival tissue from the bone defect region. Such a membrane inhibits the proliferation of soft tissue cells, including epithelial cells and fibroblasts, thereby decreasing their interference with osteogenesis and ensuring adequate spatial provision for the regeneration of periodontal tissues [267,268]. Based on their degradation feature, the GBR membranes are broadly categorized into non-absorbable and absorbable types. The non-absorbable GBR membrane is mainly fabricated from titanium alloy, which has to be removed after the reconstruction of the alveolar bone. The present absorbable GBR membrane is mainly fabricated from a polymer, whose strength is unsatisfactory to segregate gingival tissue. The most attractive feature of the absorbable membrane is no need for secondary surgery for removal [267]. Thus, the ideal absorbable GBR membrane should be characterized by excellent cytocompatibility, non-toxicity, ideal mechanical properties, superior spatial forming capacity, and optimal resorption features.

**Figure 15:**
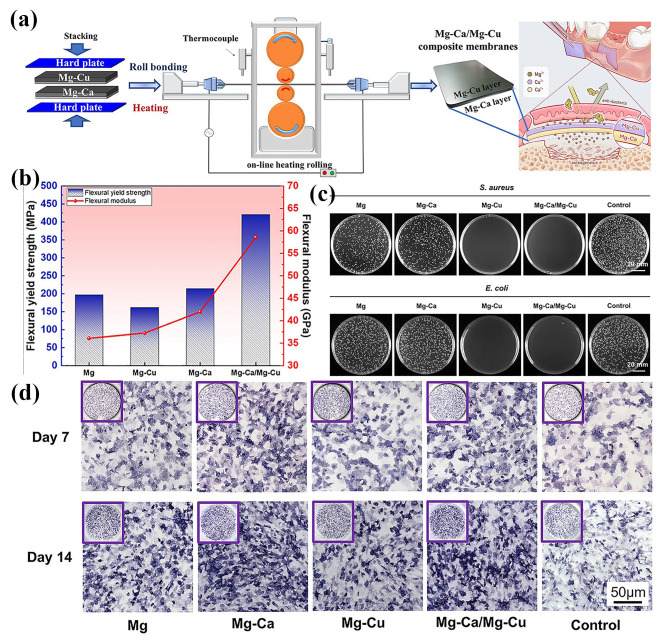
(**a**) Schematic diagram of the preparation of Mg-Ca/Mg-Cu bilayer membranes. (**b**) Bending properties. (**c**) In vitro antibacterial activity of the Mg-based bilayer membrane. Representative images exhibiting the distribution of the colony of *S. aureus* and *E. coli* after plate counting tests. (**d**) ALP staining of MC3T3-E1 cells cultured with Mg extract (Mg), Mg-Ca extract (Mg-Ca), Mg-Cu extract (Mg-Cu), Mg-Ca/Mg-Cu extract (Mg-Ca/Mg-Cu), and α-MEM medium (Control) was performed after 7 and 14 days, respectively [270].

Actually, biomedical Mg alloys have some special advantages to acting as the materials for GBR membranes [268]. Firstly, the higher strength compared to the polymer could well guarantee the bone regeneration space. Secondly, acceptable plasticity facilitates the precise shaping of the membrane for complex bone geometries. Thirdly, the alkaline corrosion products have an antibacterial effect, lowering the risk of infection. Furthermore, the bodily fluid in the alveolar bone environment has relatively low fluidity, reducing the corrosion rate of biomedical Mg alloys. The previous investigation on Mg-5Zn-0.5Zr alloys exhibited that the hot extrusion and surface fluorination could ensure its meeting the requirements of GBR membranes [267]. Such an approach enhanced its corrosion resistance, plasticity, and strength simultaneously, which reserves the space for bone and controls Mg ion release, thereby promoting osteogenesis and achieving the new bone over 80%. Rider et al. [269] conducted studies on animal models by implanting pure Mg plates, which demonstrated that Mg membranes effectively isolated soft tissues and secured the space of bone graft material. As a result, the secured space was fully replaced by newly formed bone after the ultimate degradation of the Mg plate. It is noted that the tissue regeneration outcomes are almost similar to those achieved with collagen membranes. Shan et al. [270] developed a Mg-Ca/Mg-Cu bilayer membrane by hot rolling assisted with hard plates (Figure 15a), by which the fabricated bilayer composite demonstrated outstanding flexural properties (Figure 15b). Due to the double chemical compositions, the Mg-Cu layer exhibited an attractive antibacterial effect, while the Mg-Ca layer demonstrated notable osteogenic activity (Figure 15c,d). Such a double-layer composite based on biomedical Mg alloys enlightens a new method to develop the GBR membranes. Chen et al. [271] designed the Mg-0.2Ca-0.2La alloy for the GBR membrane, which exhibited high strength and plasticity alongside excellent cytocompatibility. Moreover, it exhibited a slow degradation rate under flexural stress, indicating that it is highly suitable to act as a GBR membrane for the regeneration of intricate alveolar ridge defects. Ouyang et al. [272] demonstrated that Mg-5Ag and Mg-9Ag alloys fabricated by hot extrusion exhibited remarkable mechanical properties and antibacterial activity, demonstrating their potential to facilitate the restoration of the alveolar ridge. Si et al. [273] developed Mg-Zn-Gd alloys via extrusion and rolling, which exhibited excellent cytocompatibility and significantly promoted bone regeneration in rabbit skulls without postoperative complications.

Another critical application of the biomedical Mg alloys in dentistry lies in their utilization as a coating for dental implants. Research has demonstrated that the oral environment is highly prone to corrosion, driven by a complex interplay of individual dietary habits, the composition of oral fluids, and the presence of diverse microbial communities [274,275]. Furthermore, vertical and horizontal defects in the alveolar bone often arise due to factors such as tooth extraction, trauma, pathological conditions, or the administration of certain medications (e.g., bisphosphonates), thereby underscoring the growing clinical demand for bio-absorbable materials in bone restoration [276]. Though the human body possesses an inherent capacity for bone healing, augmenting bone mass is often imperative to ensure the successful placement of dental implants and facilitate subsequent osseointegration. Dini et al. [277] incorporated a Mg coating onto the surface of commercially pure titanium and found that the Mg-doped coating does not promote bacterial adhesion. The proliferation of MC3T3-E1 pre-osteoblasts was improved, suggesting that the Mg coating is an excellent candidate for dental implant surface treatment. The application of Mg effectively mitigates oxidative stress, minimizes periodontal tissue damage, suppresses the release of inflammatory factors, facilitates soft tissue repair, and ensures the long-term stability of the implant [275,277].

The third application of biomedical Mg alloys in dentistry is the alveolar bone fixation screw [278]. Bone preservation and primary regeneration remain critical challenges in the domain of dental medicine. The previous research showed that biomedical deformed Mg alloy fixation screws possess extensive application potential, particularly alloy incorporating strontium (Sr) and lanthanum (La), which exhibited pronounced benefits in mechanical properties, degradation kinetics, osteogenic potential, and gingival compatibility. The extruded Mg-Sr-La alloys exhibited high tensile and compressive yield strengths, twice those of pure Mg, and a relatively low degradation rate, contributing to an increased bone-implant contact area and a reduction in the occurrence of fibrous encapsulation [279]. The implantation of the Mg-Sr-La alloy in the Beagle dog model exhibited a gradual degradation feature, and its released Sr and La ions notably enhanced the expression of osteogenic markers, which promotes osteogenesis and cell migration. To enhance cytocompatibility, researchers applied an HF-treated coating on the surface of fixation screws prepared from a biomedical Mg alloy to improve the interaction with bone tissues. Histological analyses revealed that the screw with the HF-treated coating elicited a heightened macrophage response during its degradation, whereas untreated screws induced a minimal fibrous tissue response [280]. The application of HF-treated coating on the fixation screw improves its cytocompatibility, enabling its better performance in maxillofacial and dental surgical applications.

Currently, there are documented cases of NOVAMag^®^ fixation screws being utilized in clinical and experimental applications. Kačarević et al. [281] conducted an in-depth evaluation of NOVAMag^®^ fixation screws, which were fabricated from WZM211 Mg alloys with a composition of 2 wt% Y, 1 wt% Zn, and ≤1 wt% Mn (botiss biomaterials GmbH, Berlin, Germany). During the animal surgical model of GBR, the NOVAMag^®^ fixation screw exhibited favorable performance, characterized by an optimal degradation rate, robust mechanical strength, and excellent tissue compatibility, which well met the critical requirements for membrane fixation screws in GBR procedures.

The fourth important application of biomedical Mg alloys in dentistry is the additive for soft tissue regeneration. The previous study has demonstrated that Mg exerted an obvious positive influence on the repair of jaw soft tissues by effectively enhancing the proliferation, migration, and osteogenic differentiation of human dental pulp cells [282]. Furthermore, Mg has exhibited significant promotion in the migration and adhesion of human fibroblasts, facilitating the regeneration of oral mucosa. Moreover, Mg ions also enhance the healing potential of soft tissues surrounding implants by optimizing the biological characteristics of titanium implant surfaces. The releasing of alloying element ions also demonstrates an outstanding antibacterial effect, effectively suppressing the proliferation of common oral pathogens, which significantly reduces the risk of infection and thereby contributes to the recovery of soft tissues. Especially for the intricate microbial ecosystem of the oral cavity, the antibacterial effect of the biomedical Mg alloy plays a pivotal role in ensuring the healthy healing of peri-implant tissues. Early studies have revealed that biomimetic scaffolds, engineered by integrating Mg nanoparticles with polymers, significantly enhance cartilage regeneration, offering a perspective for developing soft tissue repair additives [283].

In general, biomedical Mg alloys exhibit immense potential and advantages in different stomatological applications, such as GBR membranes, dental implant coatings, alveolar bone fixation screws, and soft tissue regeneration additives. These investigations build a robust scientific foundation for more development and applications of biomedical Mg alloys in dentistry. Nevertheless, there are still some challenges, including the precise regulation of biomedical Mg alloy degradation rates and the corresponding improvement of the coating effect, which is the base for safe and efficient clinical applications. Actually, the appropriate alloy composition and processing are still the main critical issues for the biomedical Mg alloy used in dentistry, which needs the cooperation of multidisciplinary to conquer existing technical bottlenecks.

### 3.7. Tumor Treatment

The tumor is formed from an abnormal aggregation of cells arising from dysregulated cell division or abnormal proliferation, which could be categorized into benign and malignant types based on its biological properties [284,285]. The benign tumors are typically localized to their site of origin, exhibiting slow growth and well-demarcated boundaries. However, the benign tumor also has a potential risk of transforming into a malignant tumor, which needs vigilant monitoring or surgical intervention in some cases. In contrast, the malignant tumor exhibits unregulated growth, with the capacity to invade adjacent tissues (carcinogenesis) and metastasize via hematogenous or lymphatic routes to distant organs, such as the liver, lungs, brain, and bones [284]. The cancer is one of the most typical malignant tumors in the clinic. In recent years, cancer incidence has risen gradually, due to combined factors of environmental, genetic, and lifestyle. The common carcinogenic agents include biological factors (e.g., viruses), physical factors (e.g., radiation), and chemical compounds, which interact synergistically and increase the risk of cancer formation [285].

The early stage of cancer therapy mainly depends on precise detection and prompt diagnosis, which are critical preconditions [286]. For instance, Fe_3_O_4_/P/anti-E-modified Mg-based micromotors have been employed to capture circulating tumor cells (CTCs) in the bloodstream, exhibiting an innovative strategy for early cancer detection [287]. Conventional cancer therapy methods, such as surgery, radiation, and chemotherapy, often inflict substantial harm on the patient’s immune and hematopoietic systems [288]. Consequently, the pursuit of novel therapeutic measures with minimal side effects and high efficacy becomes the prominent focus in cancer research. Owing to their special biological properties, biomedical Mg alloys have attracted great attention in tumor treatment [289]. Mg has demonstrated an obvious antitumor effect against colorectal adenocarcinoma, notably inducing dose-dependent tumor cell arrest at the G0/G1 phase, suppressing cell proliferation, and initiating apoptosis. In animal experiments, the intraarticular injected Mg ions have effectively suppressed tumor tissue growth in nude mice and induced apoptosis in tumor-bearing models simultaneously [290]. The degradation products demonstrate multifaceted antitumor effects, such as suppressing tumor cell migration and invasion, inhibiting cancer-induced angiogenesis, and mitigating the formation and metastatic spread of secondary tumors [288,291,292,293,294,295]. Globig et al. [293] investigated extruded Mg and Mg-6Ag alloys and discovered that the alkaline microenvironment generated by degradation significantly suppresses osteosarcoma cell proliferation, which resulted in cellular dormancy and attenuated cancer-induced angiogenesis. Therefore, the research elucidates a potential mechanism underlying the antitumor efficacy of biomedical Mg alloy [296]. Additionally, Zan et al. [295] demonstrated that the released hydrogen (H_2_) during the degradation of biomedical Mg alloy also had antitumor effects by activating the P53-mediated lysosome-mitochondrial apoptosis signaling pathway, which was revealed through RNA sequencing and protein expression analysis. Further animal experiments revealed that inserting Mg filaments into tumor-bearing mice could inhibit tumor growth significantly via locally released hydrogen.

**Figure 16:**
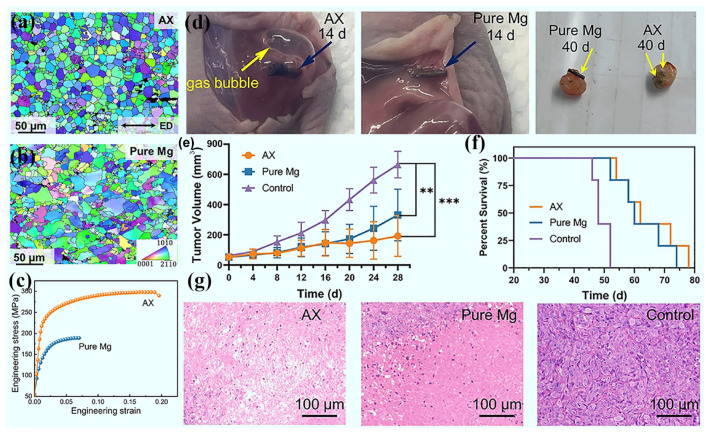
(**a**,**b**) EBSD inverse pole figure (IPF) maps of AX and pure Mg. (**c**) Tensile stress–strain curves of AX and pure Mg. (**d**) Degradation status of AX and pure Mg rods after subcutaneous implantation in mice over various time points. (**e**,**f**) Tumor growth curves and survival rates of PANC-1 tumor-bearing mice under different treatments. (**g**) H&E-stained images of tumor tissues from different treatment groups after 3–4 weeks. (Statistical significance is denoted by ** *p* < 0.1 and *** *p* < 0.01 using Student’s *t*-test) [287].

The researchers have demonstrated that the biomedical Mg alloys exhibited significant antitumor effects on diverse tumor types, such as osteosarcoma [293,296,297,298,299], breast cancer [300,301], melanoma [302], gallbladder cancer [289], colon cancer [291,295], pancreatic cancer [287], and liver cancer [287]. Recent studies mainly focus on the potential of biomedical Mg alloys (e.g., Mg-Ag [302], Mg-Gd [302], Mg-Al [287], Mg-Ag-Y [297], Mg-Al-Ca [287], WE43 [288]) as innovative agents in antitumor therapy. Specifically, Chen et al. [287] studied the Mg-6Al-0.5Ca (AX) alloy fabricated through hot extrusion, which displayed a finer and more uniform grain structure compared to pure Mg (Figure 16a,b), besides substantially enhanced yield strength and ductility (Figure 16c). The in vivo experiment revealed that the AX alloy exhibited a faster degradation rate in a mouse pancreatic cancer model, comparing to pure Mg. It effectively inhibits tumor growth by the continuously released hydrogen (H_2_), which still formed a visible bubble in the marked active region after two weeks (Figure 16d). As a result, the AX alloy and pure Mg well inhibited tumor growth, while the antitumor efficacy of the AX alloy was much better (Figure 16e–g). Moreover, RE has demonstrated remarkable potential in suppressing tumor growth. At low concentrations, RE may enhance tumor cell proliferation, while, at higher concentrations, it would inhibit proliferation and trigger apoptosis by binding specifically to DNA, by which the expression levels of oncogenes and tumor suppressor genes are modulated, thereby disrupting cellular structures and reducing lipid peroxidation [288]. Lu et al. [288] found that the presence of RE (Y, Nd, and Gd) endowed the antitumor effect to the WE43 Mg alloy that effectively induced apoptosis in tumor cells in a murine model. Despite the unique advantages of biomedical Mg alloys in antitumor therapy, there are still some challenges to conquer. It is a critical issue to realize the controllable antitumor effect in a long-term period, which needs synergistic improvement in alloying composition and corrosion behavior.

### 3.8. Biliary Stent

The biliary system is composed of the bile duct and gallbladder, which is an important organ in maintaining normal digestion by the storage, transport, and excretion of bile [303]. Biliary stricture represents a prevalent complication in hepatobiliary surgery, and the traditional treatments encompass percutaneous hepatic biliary drainage, endoscopic retrograde cholangiopancreatography (ERCP), and biliary stenting [303,304]. With the development of endoscopic technology and interventional radiology, biliary stents can be effectively implanted via endoscopic, percutaneous, or surgical approaches to alleviate biliary obstructions caused by gallstones, tumors, or inflammation. Due to its minimally invasive nature, operational simplicity, and exceptional drainage efficacy, biliary stent drainage has become the main treatment for biliary strictures [304].

**Figure 17:**
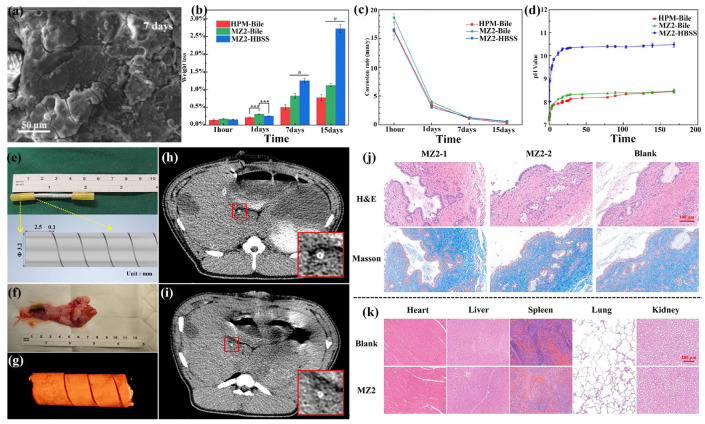
(**a**) The corrosion morphology of MZ2 in different solutions for seven days in human bile. (**b**) Weight loss (%). (**c**) Degradation rate (mm/y). (**d**) Solution pH values change. (**e**–**i**) In vivo experiment of MZ2 biliary stent implanted into pig bile duct: (**e**) MZ2 biliary stent and its design drawing, (**f**) pig common bile duct and degraded bile duct stent, (**g**) three-dimensional reconstruction of degraded bile duct stent by X-ray microscopy, and (**h**,**i**) CT image of MZ2 biliary stent after (**h**) 1 day and (**i**) 14 days implantation in vivo. (**j**) H&E staining and Masson staining of porcine bile duct epithelial tissue: MZ2-1 group is bile duct epithelial tissue close to the stent, MZ2-2 group is bile duct epithelial tissue far away from the stent, and the blank group is porcine bile duct epithelial tissue without surgery. (**k**) H&E staining of major organs of pig implanted with MZ2 biliary stent and pig without surgery [308].

The traditionally used biliary stents are primarily made from NiTi alloy and plastic. Though they could meet the basic requirements, they still have some inherent limitations. Actually, the bile exhibits a weakly acidic feature, which exerts little influence on the NiTi and plastic stents. In general, the stents are needless after their repairing function is finished, while their permanent existence would lead to rejection reactions and other complications. Consequently, the biodegradable Mg alloy stents demonstrate some attractive features for the therapy of biliary stricture [304,305,306,307]. Besides their cytocompatibility and degradability, the biomedical Mg alloys also have the advantage of releasing specific elemental ions, which could provide the bio-functions helping the recovery of the biliary tract [289,305,308]. In addition, the mechanical properties of the biomedical Mg alloys could be regulated by the alloying composition and thermomechanical processing, which meets the different requirements of patients, thereby eliminating the potential risk [289,309,310]. These advantages make biomedical Mg alloys promising candidates for biliary stents [305,306,307,308,309,310,311,312]. The research on extruded WE43 alloys exhibited remarkable corrosion resistance in bile, characterized by a notably lowered corrosion rate [311]. Further, in vivo experiments have further validated the potential applications of biomedical deformed Mg alloy stents for biliary stricture therapy. For example, Peng et al. [308] investigated the degradation behavior of high-purity Mg and MZ2 alloys fabricated by hot extrusion and rolling in human bile, which indicated that an oil-like structure was formed on the degradation product surface layer of the MZ2 alloy (Figure 17a). After long-term immersion, the MZ2 alloy demonstrated a slower degradation rate in bile, accompanying with a gradual increase in pH value (Figure 17b–d). Implantation experiments involving MZ2 alloy biliary stents in porcine common bile ducts revealed that the overall contour remained clearly visible after two weeks (Figure 17e–i). The Masson staining results demonstrated that the bile duct tissue structure remained intact, and the wounds resulting from stent implantation had completely healed (Figure 17j). The histological examinations of major organs, including the heart, liver, spleen, lung, and kidney, revealed no abnormalities (Figure 17k). These findings suggest that the implantation of MZ2 alloy biliary stents and their subsequent degradation within the bile ducts do not induce adverse effects on major organs. Moreover, the common bile duct with an implanted MZ2 alloy biliary stent exhibited neither blockage nor narrowing over the observation period, and bile drainage functionality remained normal. Liu et al. [313] observed that the AZ31 stent, when implanted into the rabbit common bile duct for six months, underwent gradual degradation, with post-surgical inflammation levels returning to normal over time. Zhang et al. [314] designed the ZX20 biomedical Mg alloy braided biliary stent, which could well meet the clinical requirements by optimizing radial compressive strength and implantation duration.

Notably, the UNITY-B balloon-expandable biomedical Mg alloy biliary stent, developed by Q3 Medical Devices, has received CE certification and emerged as a vital option for managing pancreaticobiliary obstruction [315]. UNITY-B, composed of a novel Mg alloy (MgNdMn21) and coated with a polymer layer, is structurally engineered to mimic coronary drug-eluting stents (DESs). Clinical trials have demonstrated its robust safety and efficacy, achieving a success rate of 94.4% [315,316,317,318]. Specifically, the biodegradability of biomedical Mg alloys could endow more customized functions by the alloying composition design and processing regulation. However, adaptability is still an important factor, which needs systematical optimization on the biliary stent fabricated by the biomedical Mg alloys.

As a kind of novel material, Mg and its alloys have attracted much attention in recent years. Especially for biomedical Mg alloys, their exceptional and controllable properties have demonstrated great potential application in many fields. However, the disadvantages of biomedical Mg alloys such as insufficient strength and relative rapid degradation could not well meet the requirement of implanting in the human environment. So many studies have been performed to increase the mechanical properties and corrosion resistance, which promotes the development of biomedical Mg alloys and their clinical applications [34,319,320].

**Figure 18:**
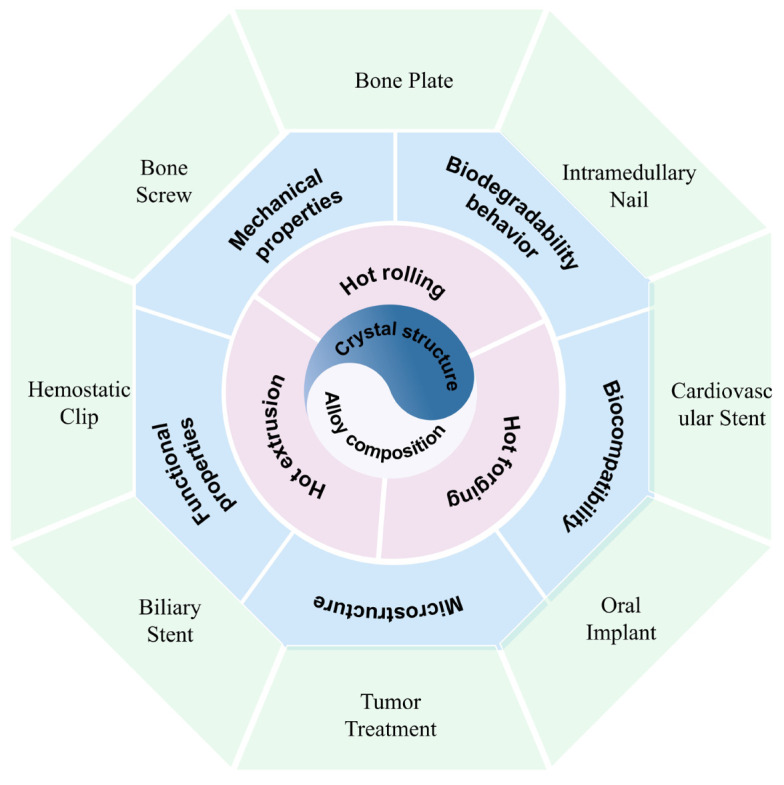
The schematic diagram of the relationship between deformation processing and performance of biomedical Mg alloy.

For the Mg alloy, the hexagonal crystal structure of the Mg matrix restrains the activity of the slipping system, which has been thought of as the main obstacle to its processing and mechanical properties. Its chemical activity benefits bio-degradation but leads to uncontrollable evolution, especially in the medium stage. Through the main elements, such as Mg, Zn, and Ca, which are the major or trace elements essential to the human body, they have the acceptable daily intake (Mg: ~330 mg/d, Zn: 12.5 mg/d, Ca: 800 mg/d). Meanwhile, for the RE elements, their acceptable daily intake is 4.41 mg/d [57]. Their excessive releasing from degraded Mg alloys would result in local biotoxicity, which restrains the tissue from reconstructing and healing. Generally, the performance of a metal or alloy is determined by its microstructure, phase constituent, precipitation morphology and distribution, crystal defect, and stress [321,322]. Then, it becomes a critical issue to regulate the microstructure of biomedical alloys by alloying and thermomechanical processing. The optimization routes for Biomedical Mg alloys and their application can be summarized in Figure 18. The alloy composition is the fundamental factor, which determines the phase constituents and crystal structure. Most alloying elements mainly act as the inducements of secondary phases but have little influence on the matrix. The Li addition with a high ratio could prompt the phase with a cubic crystal structure and change the deformation behavior, producing high ductility. The reasonable addition of RE elements could enhance corrosion resistance by enhancing electrode potential and forming densified corrosion products. Moreover, the appropriate alloying elements, such as Sr, Zn, and Mn, could realize the specific functions, such as antibacterial effect, osteogenesis promotion, anti-inflammatory, etc. The thermomechanical processing of hot forging, hot rolling, and hot extrusion reconstructs the morphology of the grain and secondary phase, increasing mechanical properties significantly. The grain refinement improves strength and ductility by handicapping dislocation movement and retraining stress concentration. Due to the refinement of the secondary phase, the galvanic corrosion could be well weakened, which benefits the homogeneous degradation and decreases the degradation rate. Such an improvement enhances the cytocompatibility of the biomedical Mg alloy obviously. It can be summarized that the synergistic cooperation of alloying and thermomechanical processing optimizes the microstructure, mechanical properties, corrosion behavior, and cytocompatibility of the biomedical Mg alloys, which contributes to their applications as implants, such as cardiovascular stent, intramedullary nail, bone plate, bone screw, hemostatic clip, biliary stent, oral implants, and tumor therapy. In particular, the requirements for biomedical Mg alloys vary significantly depending on the specific clinical application. Mostly, the high strength and low degradation rate are the main requirements for biomedical Mg alloys, especially for stents and bone implants. For oral implants and tumor therapy, functional ion releasing is the basic demand, while the stable ion releasing rate is important for this kind of implant, which determines the functional effectiveness. The safety and effectiveness of clinical implants require better performance, which indicates that more efforts should be taken to improve the biomedical Mg alloy further.

## 4. Conclusions

Biomedical Mg alloys have attracted much attention, due to their special performance such as high specific strength, low density, low elasticity modulus, high damping capacity, biodegradation, relatively good cytocompatibility, etc. However, the low corrosion resistance and insufficient strength also bring many challenges to their clinical application.

For the biomedical Mg alloy, the alloying composition plays a fundamental effect on its mechanical properties and corrosion behavior, while the thermomechanical processing could exert an important effect. To achieve optimal performance, the biomedical Mg alloy should be carefully designed from fundamental alloying composition to the corresponding thermomechanical processing, meeting the implant requirements in specific clinical applications. The rational and non-toxic alloying element selection is the basic premise for the biomedical Mg alloy, which determines the bio-safety of its products. However, they should take full use of the solid solution and precipitation strengthening effect, making them beneficial to the subsequent deformation processing. In general, the Zn, Ca, and Mn are the preferential alloying elements, while the trace (0.5~3%) RE (Y, Nd, Ce, La, Gd, Dy) addition could be considered, because of their significant improving effect. Sometimes, the alloying strengthening conflicts with the bio-safety, which requires a good balance between them. The bio-safety is the primary choice for biomedical Mg alloys.

The thermomechanical processing technique plays an important role in the microstructure and performance of the biomedical alloy, which determines the final performance of its implants. Due to the rheological deformation in a specific direction, the single thermomechanical processing always results in obvious textures in the deformed biomedical Mg alloys, which influences the homogeneity in microstructure and performance. Therefore, it is a critical issue to set reasonable thermomechanical processing and parameters for the specific biomedical Mg alloy. The combination of different thermomechanical processing techniques sequentially would be a better choice for biomedical Mg alloys. In practice, the hot extrusion would be preferred for the initial processing, due to its excellent adaptability; while the hot rolling and hot forging could be chosen for the final processing to further improve mechanical properties.

Because of the diversified requirements of different clinical implants, the performance of the biomedical Mg alloys should be varied. The higher strength and lower biodegradation rate are the main research objective, thereby becoming the research hot spots for the biomedical Mg alloy. Though the present Mg alloy using specific thermomechanical processing could meet most requirements, there are always some non-ideal aspects. For biodegradable implants, the best strategy is designing the biomedical Mg alloy based on the clinical application while not optimizing the industrial Mg alloy to meet the clinical requirements. Combined with the present abundant studies, the design of better biomedical Mg alloys with specific performance would be the main focus in the future, which could fulfill the clinical requirements better.

## Figures and Tables

**Table 1:** Advantages of alloying elements in biomedical Mg alloys and their effects on alloy performance.

Alloying Element	Biological Benefits	Performance Benefits	Reference
Zn	Non-cytotoxic element, good cytocompatibility	Grain refinement, increasing corrosion resistance and mechanical properties.	[65]
Mn	Low hemolysis rate, regulating immunity effect, and good cytocompatibility	Increasing corrosion resistance and strength.	[66]
Ca	Essential bone component, good cytocompatibility, and anticarcinogenic function	Decreasing corrosion resistance, increasing strength and ductility.	[67]
Sr	Osteogenesis promotion and good cytocompatibility	Grain refinement, increasing corrosion resistance.	[68]
Sn	Essential trace element of human body, inducing tissue regeneration and good cytocompatibility	Improving corrosion resistance.	[69]
Li	Nutrient element for central nervous system and good cytocompatibility	Increasing specific strength, and promoting the activity of slip system.	[21]
Y	Relative good cytocompatibility at low content	Grain refinement, promoting the activity of non-basal slip systems, improving ductility and corrosion resistance.	[70]
Nd	Relative good cytocompatibility at low content	Grain refinement, precipitation strengthening, improving corrosion resistance, but detrimental to ductility.	[70]
Ce	Relative good cytocompatibility at low content and osseointegration	Grain refinement, precipitation strengthening but detrimental to corrosion resistance.	[71]
Gd	Relative good cytocompatibility at low content	Grain refinement, precipitation strengthening, but detrimental to ductility.	[72]
Zr	Relative good cytocompatibility at low content	Grain refinement, increasing strength and ductility.	[72]
Dy	Relative good cytocompatibility at low content	Grain refinement, promoting the activity of non-basal slip systems, improving ductility and corrosion resistance.	[73]

**Table 2:** Summary on advantages and disadvantages of widely applied thermomechanical processing techniques for biomedical Mg alloys.

Thermomechanical Processing	Advantages	Disadvantages	Reference
Conventional extrusion (Direct extrusion)	(1) Grain refinement; (2) Wide applicability; (3) Reduction in metallurgical defects; (4) Improvement in compactness; (5) High surface quality.	(1) Limited billet length; (2) Prone to porosity, cold separation, poor filling, cracking, and cold interlayers; (3) High sensitivity to temperature control; (4) Uneven deformation.	[88]
Equal-channel angular pressing	(1) Grain refinement; (2) Improved microstructure uniformity; (3) Significant improvement in mechanical properties; (4) Formation of nanocrystalline.	(1) Limited billet size; (2) Limited application in alloys; (3) Extremely high crystal defects and possible damage.	[89,90]
Cyclic extrusion compression	(1) Grain refinement; (2) Improvement in mechanical properties; (3) Obtaining ultra-fine grains and uniformly distributed secondary phase; (4) Continuous processing.	(1) Accurate reverse compression parameters; (2) Having critical passes and minimum grain size; (3) Cracking occurs in exceeded critical passes.	[91,92,93,94]
Conventional rolling	(1) Grain refinement; (2) High production efficiency; (3) Improved mechanical properties; (4) Good uniformity; (5) Wide range of applications.	(1) Uneven deformation; (2) Low product accuracy; (3) Stress concentration issues; (4) Poor deformation coordination, wrinkling, low secondary formability, and high cracking susceptibility.	[2,95,96,97]
Cross-rolling	(1) Homogenization of mechanical properties; (2) Reduction in internal stress; (3) Grain refinement; (4) Improvement in the surface quality; (5) Reduction in cracks and defects.	(1) Complex process control; (2) Limited scope of application; (3) Potential introduction of defects; (4) Low production efficiency.	[98]
Accumulative roll bonding	(1) Ultra-fine microstructure; (2) Diversified applications; (3) Capability to process large billet; (4) High stability.	(1) Interlayer bonding issues, local necking, and fragmentation; (2) Uneven thickness distribution across layers; (3) Non-uniform dislocation slip; (4) Complex process.	[99,100,101,102]
Conventional forging	(1) Grain refinement; (2) Industrialized mass processing; (3) Fewer processing defects.	(1) Cracks and localized excessive deformation; (2) Limited shape complexity; (3) Uneven microstructure.	[82,103]
Multi-directional forging	(1) Fewer processing defects; (2) Suitable for large-size and complex-shaped workpieces; (3) Improved product quality; (4) Enhanced isotropy; (5) Reduced residual stress.	(1) Complex equipment and process control; (2) Limited scope of application; (3) Relatively low production efficiency; (4) Potential for local deformation of the material.	[104,105,106]
Radial forging (Rotary swaging)	(1) Grain refinement; (2) High material utilization; (3) Enhanced microstructure uniformity; (4) Low residual stress; (5) High machining accuracy.	(1) Complex process control; (2) Limited shape adaptability; (3) Unstable billet flow; (4) High billet size requirements.	[82]

**Table 3:** Mechanical properties of typical biomedical Mg alloys processed via different thermomechanical processing.

Alloys	Processing	Yield Strength (MPa)	Tensile Strength (MPa)	Elongation (%)	Reference
Mg-3Zn-0.2Ca	HE	215.9	270.2	11.14	[50]
Mg-Zn-Ca-Mn	HR	146	229	1.6	[60]
Mg-2Zn-0.7Ca-1Mn	HE	229	278	10	[66]
Mg-0.8Ca	MDF	199	264	9.4	[67]
Mg-1Zr-0.5/1Sr-0.5/1/1.5/2Dy	HE	163.7–231.8	229.4–258.3	11.6–23.9	[80]
Mg-2.22Zn-2.25Ga	HE	128	261	22.0	[176]
Mg-2.9Zn-1.1Ca-0.5Mn	HE	352.5	382.3	7.1	[107]
Mg-1.03Zn-0.66Ca (ZX 11)	RS	210	276	18.3	[117]
Mg-1Zn-0.5Sn	HE	115	239	\	[122]
Mg-2Zn-0.6Zr-0.6Nd	HE	242–269	274–298	25.6–26.1	[121]
Mg-4Zn-4Ga	HE	256	343	14.2	[66]
Mg-2.0Zn-1.6Ca	HE	/	283.47–393.96	10.84–18.08	[189]
Mg-4/6Zn-0.6/0.8Y-0.5Nd	HE	153–252.6	245.6–308.8	10.0–20.2	[123]
Mg-2Zn-0.46Y-0.5Nd	HE	139.4	249.4	21.1	[186]
Mg-1.5Y-1.2Zn-0.44Zr	HE	178	236	28	[190]
Mg-4Zn-0.8Y-0.5Nd	HE	252.6	308.8	10.0	[191]
Mg-0.035/0.5/1/3Zr-0.2/0.5/1/3Sr	HE	210–275	253–302	5.9–11.1	[192]
Mg-2Zn-0.6Ca-1Er	HE	128	225	17.2	[117]
Mg-3Nd-0.5Zn	HE	251–337	271–338	0.5–5.9	[193]
Mg-3Zn-0.5Sr	HE	164	254	19	[194]
Mg-3Zn-0.5Sr-0.2/0.5Ca	HE	126–185	257–305	15–29	[194]
Mg-4.12Y-2.15Nd-0.43Zr-0.26La	ECAP + Ex	225–350	325–398	9.45–12.20	[195]
Mg-0.24Sn-0.04Mn	HR	117.9	178.8	9.1	[196]
Mg-0.24Sn-0.04Mn	HE	129	219.6	7.9	[196]
Mg-0.24Sn-1.16Zn-0.04 Mn	HR	178	223	5.2	[196]
Mg-0.24Sn-1.16Zn-0.04 Mn	HE	142.3	269.2	16.5	[196]

Rotary swaging (RS), hot extrusion (HE), hot rolling (HR), and multi-directional forging (MDF).

**Table 4:** Corrosion rates of biomedical Mg alloys prepared by different thermomechanical processing.

Alloys	Processing	Corrosion Medium	Corrosion Rate (mm/y)	Reference
Mg-1Zr-0.5Sr-1Dy	HE	SBF	3.37	[80]
Mg-4Zn-0.5Ca-0.75Mn	HE	SBF	0.12	[200]
Mg-1.03Zn-0.6Ca	RS	FBS	0.44	[117]
Mg-1Zn-0.1Ca	HE	SBF	3.2	[197]
Mg-0.45Zn-0.45Ca	HE	α-MEM medium	1.04	[201]
Mg-1Zn-0.3Ca	HE	Right tibia of sheep	0.27	[202]
Mg-2Sr-Zn	HR	HBSS	0.85	[68]
Mg-2Sr-Ca	HR	HBSS	1.10	[68]
Mg–2Sr	HR	HBSS	1.37	[68]
Mg-3Zn-0.2Ca-0.5Y	HE	SBF	5	[49]
Mg-2Zn-0.7Ca-1Mn	HE	HBSS	0.3	[203]
Mg-1.0Zn-0.3Ca	HE	SBF	0.091	[204]
Mg-1.5Zn-0.25Ca	HE	SBF	0.123	[204]
Mg-3.0Gd-2.7Zn-0.4Zr-0.1Mn	HE	HBSS	0.46 ± 0.19	[205]
Mg-2Zn-0.6Ca-1Er	HE	0.5 wt.% NaCl solution at 25 °C	7.83	[206]
Mg-2Zn-0.6Ca-1Er	HE	PBS at 37 °C	1.55	[206]
Mg-0.5Zn-0.35Zr-0.15Mn-2Tb	HE	HBSS	0.10	[207]
Mg-0.24Sn-0.04Mn	HR	HBSS	0.51	[196]
Mg-0.24Sn-0.04Mn	HE	HBSS	0.71	[196]
Mg-0.24Sn-1.16Zn-0.04 Mn	HR	HBSS	2.87	[196]
Mg-0.24Sn-1.16Zn-0.04 Mn	HE	HBSS	0.95	[196]
Mg-6Zn	MDF	0.1 M NaCl solution	0.34	[65]

## Data Availability

No new data were created or analyzed in this study.
